# Comparing apples and oranges: Why infant bone collagen may not reflect dietary intake in the same way as dentine collagen

**DOI:** 10.1002/ajpa.23682

**Published:** 2018-09-06

**Authors:** Julia Beaumont, Elizabeth‐Craig Atkins, Jo Buckberry, Hannah Haydock, Pennie Horne, Rachel Howcroft, Kevin Mackenzie, Janet Montgomery

**Affiliations:** ^1^ School of Archaeological and Forensic Sciences University of Bradford Bradford West Yorkshire United Kingdom; ^2^ Department of Archaeology University of Sheffield Sheffield South Yorkshire United Kingdom; ^3^ Centre for Archaeology and Anthropology Bournemouth University Dorset United Kingdom; ^4^ Institute of Medical Sciences University of Aberdeen Aberdeen United Kingdom; ^5^ Department of Archaeology University of Durham Durham United Kingdom

**Keywords:** in utero, maternal health, physiological stress, stunting, weaning

## Abstract

**Objectives:**

Recent developments in incremental dentine analysis allowing increased temporal resolution for tissues formed during the first 1,000 days of life have cast doubt on the veracity of weaning studies using bone collagen carbon (δ^13^C) and nitrogen (δ^15^N) isotope ratio data from infants. Here, we compare published bone data from the well‐preserved Anglo‐Saxon site of Raunds Furnells, England, with co‐forming dentine from the same individuals, and investigate the relationship of these with juvenile stature. The high‐resolution isotope data recorded in dentine allow us to investigate the relationship of diet with juvenile stature during this critical period of life.

**Materials and methods:**

We compare incremental dentine collagen δ^13^C and δ^15^N data to published bone collagen data for 18 juveniles and 5 female adults from Anglo Saxon Raunds Furnells alongside new data for juvenile skeletal and dental age. An improvement in the method by sampling the first 0.5 mm of the sub‐cuspal or sub‐incisal dentine allows the isotopic measurement of dentine formed in utero.

**Results and discussion:**

δ^13^C profiles for both dentine and bone are similar and more robust than δ^15^N for estimating the age at which weaning foods are introduced. Our results suggest δ^15^N values from dentine can be used to evaluate the maternal/in utero diet and physiology during pregnancy, and that infant dentine profiles may reflect diet PLUS an element of physiological stress. In particular, bone collagen fails to record the same range of δ^15^N as co‐forming dentine, especially where growth is stunted, suggesting that infant bone collagen is unreliable for weaning studies.

## 1 INTRODUCTION

An evaluation of the breastfeeding and weaning behavior of past populations using the stable isotope ratios of carbon (δ^13^C) and nitrogen (δ^15^N) from the body tissues as a method of estimating diet continues to be the subject of many studies. Because infant feeding practices have varied throughout human history as a response to cultural and environmental change, and because breastfeeding (and the lack of breastfeeding) can affect the health of both mother and infant, the interpretations have been used to investigate factors such as rates of infant mortality, birth‐spacing, and maternal occupation (for a summary, see Tsutaya & Yoneda, 2015).

Many studies use isotope ratio measurements of bone collagen δ^13^C and δ^15^N from infants of different ages at death on the grounds that these data will represent the diet of each individual during life. These data are then compared to bone collagen data from females of child‐bearing age within the same population (e.g., Jay, Fuller, Richards, Knüsel, & King, 2008) to identify mother/infant trophic level shift, which has been demonstrated in the tissues of modern mother/infant pairs (de Luca et al., 2012; Fogel, Tuross, & Owsley, 1989; Fuller, Fuller, Harris, & Hedges, 2006). However, because of factors such as slow turnover of bone in both mother and infant, and the unknown effect of any disease or nutritional stress on the δ^15^N values of infants who have died, the assumption that the data from infant bone collagen accurately reflect diet in the individual, and can be used as a proxy for the population as a whole, seems increasingly unsafe (Beaumont, Montgomery, Buckberry, & Jay, 2015; DeWitte & Stojanowski, 2015). The use of incremental dentine collagen to assess the childhood diet of both infants who died and adults who survived their early years has produced isotope profiles which can show detailed temporal changes in the isotope ratios: moreover, the magnitude of δ^15^N values can be related not only to dietary change but also to periods of physiological stress (Armit, Shapland, Montgomery, & Beaumont, 2015; Beaumont et al., 2015; Henderson, Lee‐Thorp, & Loe, 2014; Montgomery et al., 2013). During periods of undernutrition, the body can enter a catabolic state during which an individual will use amino acids from their own body tissues to synthesise new proteins such as collagen. This will have the effect of increasing the δ^15^N values in the same way as a trophic level shift (and see discussion in Katzenberg & Lovell, 1999). A recent publication has also shown the relationship between the isotope ratios in breastmilk and the maternal and infant fingernails, albeit in a single modern pair (Herrscher, Goude, & Metz, 2017). This provides evidence for the stability of the δ^15^N values in breastmilk in this well‐nourished pair, with a smaller than expected shift in δ^15^N between maternal and infant fingernails. However, there are significant changes in the δ^13^C of breastmilk which decreased over the period of breastfeeding, and which could be related to the increase in storage of fat during pregnancy and recycling of maternal fat stores during breastfeeding. Fat stores are built up during the first two trimesters to be available for the fetus during the third trimester (Butte, Hopkinson, Wong, Smith, & Ellis, 2000) which may in turn alter the mother's δ^13^C. Cameron (2012) reports a study of 36 fetuses which found a dramatic increase in the average weight of fat between 30 and 40 weeks gestation from 30 g to 430 g, interpreted as a high‐energy store for the post‐partum period. Where fat stores have been recycled this produces a fall in the δ^13^C as described by Neuberger, Jopp, Graw, Püschel, and Grupe (2013), Lehn, Rossmann, and Graw (2015), and Cherel, Hobson, Bailleul, and Groscolas (2005) and which was seen in the dentine collagen of juveniles from Kilkenny workhouse during starvation (Beaumont & Montgomery, 2016).

A recent study by Beaumont, Gledhill and Montgomery (2014) has shown that the δ^13^C and δ^15^N values of human dentine collagen can be measured either using the denatured and lyophilized (freeze‐dried) product, or by freeze‐drying a smaller section of the demineralized collagen. In that study, each dentine section was divided into two: one portion which was denatured/lyophilized and a second which was only frozen and freeze‐dried prior to analysis. The δ^13^C and δ^15^N values of the two differently‐treated portions of the section are comparable, and the quality parameters for the *C*:*N* ratio remained within the limits deemed acceptable by DeNiro (1987) even when using dentine from teeth where the preservation was poor (Beaumont et al., 2014). This means that a much smaller sample than previously can be reliably used to measure δ^13^C and δ^15^N and opens the way for sampling of the δ^13^C and δ^15^N of incremental dentine with even greater temporal resolution, enabling a detailed analysis of perinatal diet, breastfeeding and weaning.

It has been a feature of some isotope studies that data from both dentine and bone collagen have been used interchangeably (e.g., (King et al., 2018; Müldner, Chenery, & Eckardt, 2011; Sandberg, Sponheimer, Lee‐Thorp, & Van Gerven, 2014). However, because of the uncertainty about the temporal period represented by the bone collagen, it is critical that we understand how the two are related: whilst it may be valid to compare dentine and bone collagen when investigating juvenile and adult data from the same individual, do both tissues record the same values at the same period of life? Data from 19th century Lukin Street, London (Beaumont, 2013) has shown that some infants have no overlap between the deciduous incremental dentine profiles and bulk bone collagen δ^15^N, meaning that even bulk dentine collagen and bone collagen would differ.

This study is the first time that a previously‐published bone collagen δ^13^C and δ^15^N analysis of breastfeeding and weaning has been re‐investigated using the incremental dentine from the same individuals. The skeletal remains of the individuals in question derive from the Anglo‐Saxon cemetery site of Raunds Furnells and are curated at the Biological Anthropology Research Centre, University of Bradford. The human remains from this site have been the subject of a number of anthropological studies (Craig, 2005; Craig & Buckberry, 2010; Hoppa, 1992; Lewis, 2002; Powell, 1996, 113–124) including previous investigation of δ^13^C and δ^15^N (Haydock, Clarke, Craig‐Atkins, Howcroft, & Buckberry, 2013; Howcroft, 2008). In the latest study by Haydock et al. (2013), a significant quantity of δ^13^C and δ^15^N data were produced from bone collagen samples taken from 20 adults and 59 children. The site has been the subject of intensive research interest because of the large number of burials and good bone preservation of the individuals excavated (*n* = 361), of which a substantial proportion are the unusually well‐preserved remains of juveniles (*n* = 162; Boddington, 1996; Craig & Buckberry, 2010; Hadley & Buckberry, 2005). Raunds Furnells also benefits from a well‐understood site chronology. Radiocarbon dates for the graveyard give a combined date range of cal AD 978–1040 to two sigma (Boddington, 1996, 72); thus, it is apparent that the cemetery was founded in the 10th century and went out of use before the Norman Conquest (AD 1066).

The isotope data from Howcroft (2008) and Haydock et al. (2013) are consistent with other contemporary inland Anglo‐Saxon sites and suggest a diet mainly composed of C_3_ plants and terrestrial animals, with some input from freshwater fish (Mays & Beavan, 2012).

### 1.1 Nutrition and growth

In a healthy population, growth and height is primarily determined by genetics, but can also be influenced by the environment, nutrition, and socioeconomic status (Floud, Wachter, & Gregory, 1990; Hoppa, 1992; Larsen, 2015; Mays, 2016; Sutphen, 1985). Growth velocity (the rate of change of stature) is considerably greater during the first 6 months of life, before it settles to a gradual increase in height per year (Lejarraga, 2012). The cessation or slowing of long‐bone growth can result in a decrease in the potential stature of the individual, known as stunting (Saunders & Hoppa, 1993; WHO, 2013). This could result in a permanent small stature, particularly in females (Bose, 2018), although Tanner held the view that “the undernourished child slows down and waits for better times” (Tanner, 1989,130). Human growth has been shown to occur in a saltatory pattern: short intermittent episodes which vary by anatomical site. Each person will have individual episodes of measurable growth (saltations) punctuated by periods of no growth (stasis) the patterns of which are mediated by genetic and environmental factors. The final stature of the individual is the accumulation of different frequency of saltations and the amount of growth during each event (Lampl, 2012). This allows for “catch‐up growth” if conditions improve. Stature has been shown to be sensitive to both environmental conditions (such as nutrition and disease) and physiological factors (Jantz & Owsley, 1984) and stunted growth is one of the main complications that can result from chronic inflammation and infection in juvenile individuals (Pinhasi, Teschler‐Nicola, Knaus, & Shaw, 2005). In contrast, dental development is not significantly affected by environmental impacts. It has, for example, been demonstrated that no form of malnutrition—neither acute nor chronic—has any measurable impact on the timing of tooth formation (Elamin & Liversidge, 2013; Ives, 2015; Lewis, 2007, 38). Thus, a comparison of skeletal age and dental age in juveniles can be used as a measure of environmental stress, as discussed in Mays, Brickley, and Ives (2008). The Raunds Furnells individuals have been the subject of several previous studies which examined osteological markers of biological stress including Harris lines, enamel hypoplasia, cribra orbitalia, and porotic hyperostosis (Craig & Buckberry, 2010; Haydock et al., 2013; Lewis, 2002; Ribot & Roberts, 1996). These studies conclude that this population experienced high levels of biological stress during their lives.

## 2 MATERIALS AND METHODS

Eighteen juveniles were selected from those previously analyzed to investigate δ^13^C and δ^15^N variations in bone collagen data by Haydock et al. (2013) and Howcroft et al. (2012) representing a range of ages at death and δ^15^N bone collagen values. Five females with age‐at‐death estimates of young adult (18–25 years) or young middle adult (25–35 years) were selected to represent the childhood values of the putative mothers whose δ^15^N bone collagen values were used in the plots produced by Howcroft et al. (2012)(see Table 1). The femora and dentition of 15 juveniles from the sample of 59 in the study by Haydock et al. (2013; including 8 of the 18 selected for the incremental dentine analysis in this study) were complete enough to allow the re‐assessment of skeletal and dental age.

**Table 1:** Tooth notation, developmental stage of teeth, and age at death of individuals from Raunds‐Furnell

Site	Skeleton number	Tooth notation	Tooth selected	Estimated skeletal age in years
RAUNDS	5251	61	UL DI1	0.0
RAUNDS	5082	71	L DI1	1.3
RAUNDS	5109	51	UR DI1	2.0
RAUNDS	5140	61	UL DI1	2.0
RAUNDS	5273	54	UR DM1	2.0
RAUNDS	5012	61	UL DI1	2.5
RAUNDS	5102	82	LR DI2	2.5
RAUNDS	5292	52	UR DI2	2.5
RAUNDS	5005	85	LR DM2	3.0
RAUNDS	5023	71	LL DI1	3.0
RAUNDS	5264	74	LL DM1	3.5
RAUNDS	5345	55	UR DM2	3.5
RAUNDS	5354	54	UR DM1	3.5
RAUNDS	5170	84	LR DM1	4.0
RAUNDS	5212	73	LL DC	4.0
RAUNDS	5070	61	UR DI1	5.0
RAUNDS	5135	65	UL DM2	5.5
RAUNDS	5338	26	ULM1	6.0
RAUNDS	5125	65	UL DM2	7.0
RAUNDS	5154	16	UR M1	18–25
RAUNDS	5187	41	LRI1	18–25
RAUNDS	5021	41	LR I1	25–35
RAUNDS	5093	12	UR I2	25–35
RAUNDS	5239	42	LR I2	25–35

### 2.1 Measuring in utero dentine development

**Figure 1:**
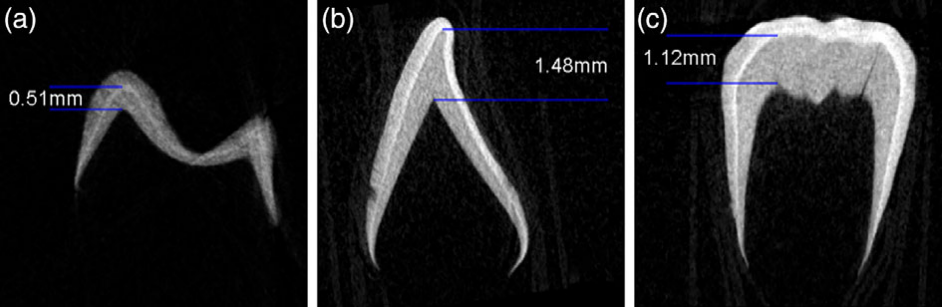
Micro‐CT images slices of upper second deciduous molar, coronal view (a), and coronal view (b) and sagittal view (c) of lower left first deciduous incisor from individual 469 from the stack collection (Royal College of surgeons England) aged 40.5 weeks gestation. Measurements in mm are shown of the depth of dentine developed in the cuspal (a) and incisal (b and c) areas [Color figure can be viewed at http://wileyonlinelibrary.com]

Micro‐CT scanning of deciduous teeth from modern individuals of known‐age from the Stack collection (Wellcome collection at the Royal College of Surgeons, London) allowed the measurement of the depth of dentine which had developed before death. These measurements showed that all deciduous teeth had formed at least 0.5 mm of dentine prior to 40 weeks gestation, thus this first‐forming section of dentine is considered to represent pre‐natal δ^13^C and δ^15^N among the archaeological sample in this study (Figure 1). Scanning was carried out at the Microscopy and Cellular Imaging Facility, University of Aberdeen using a SkyScan‐1,072 high‐resolution desk‐top micro‐CT system. Teeth were scanned at 100 kV/98.4uA at a magnification of ×23, giving a pixel resolution of 13.31 μm. Back projection images were reconstructed using NRecon software, viewed in Dataviewer and shown in greyscale (Figure 1).

### 2.2 Stable isotope analysis

A single tooth was taken from each of the 18 juveniles and 5 adults. This was a deciduous tooth for 17 of the juveniles, permanent first molar (M1) for the remaining juvenile individual (for whom no deciduous teeth were available). Deciduous teeth, and most M1 teeth, begin to form before 40 weeks gestation (AlQahtani, Hector, & Liversidge, 2010). The deciduous teeth grow during the first 2.5 years of life, thus recording the isotope ratios from in utero to early childhood and acting as an archive of these where present in juveniles. A permanent incisor or M1 tooth was sampled from each of the adults and was chosen based on the lack of wear on the incisal/cuspal tip (see Table 1). The permanent M1 continues to form until the age of 8.5 years ±0.5 years. Permanent incisors begin to form in the first 3–6 months of life and continue to grow until 6–10 years (depending on the tooth). Thus, these teeth represent tissue from the earliest period of childhood available in the older child or adult (AlQahtani et al., 2010; AlQahtani, Hector, & Liversidge, 2014). In some cases, the teeth were still developing, or some of the deciduous teeth had undergone some root resorption prior to death, which reduces the amount of the life course available to measure.

Each tooth was cleaned by air abrasion, a single root removed from molar teeth and incisor teeth were bisected, and the bulk of the enamel removed from the sampled portion using a hand‐held saw. There were no macroscopically visible areas of caries or secondary dentine present on any of the teeth sampled, thus avoiding the measurement of damaged collagen or tissue which has grown later than the primary dentine (Beaumont, Gledhill, Lee‐Thorp, & Montgomery, 2013).

Each tooth sample was demineralized in 0.5 M HCl at 4 °C following the modified Longin method (Brown, Nelson, Vogel, & Southon, 1988) and then sectioned according to the second method in Beaumont et al. (2013) using a scalpel.

For each of the juveniles, the first‐forming 0.5 mm was removed from the incisal edge/cusp tip as a first sample, and 1 mm samples taken thereafter down the length of the tooth.

The first 0.5 mm dentine sample of each deciduous tooth was frozen, freeze‐dried, and measured without denaturing (see above and Beaumont et al., 2014). The rationale for analyzing this smaller sample separately is that only the first 0.5 mm of tissue forms before birth, and therefore contains isotope ratio values from the in utero period. Because human dentine is laid down in an overlapping pattern, incremental horizontal sampling of the tissue throughout most of the tooth results in averaging of the isotopic values attenuation of the signal. However, as there is little overlap of the layers nearest to the enamel‐dentine junction, this attenuation will be reduced in the earliest‐forming dentine, giving a more accurate result.

All of the 1 mm demineralized dentine sections were denatured by heating to 70 °C in a pH 3 solution of HCl for 24 hr, frozen and then freeze‐dried.

Each of the samples was analyzed in duplicate. The samples were combusted in a Thermo Flash EA 1112 and the separated N_2_ and CO_2_ was introduced to a Delta plus XL via a Conflo III interface. All samples were interspersed with laboratory and international standards, and the analytical error was determined to be 0.2‰ or less.

### 2.3 Assigning dental and skeletal age

In the original isotopic studies by Howcroft (2008) and Haydock et al. (2013), the completeness of the individuals was not a factor in the selection of samples. Thus, only eight of the juveniles in this study sampled for incremental dentine were sufficiently well‐preserved to allow skeletal and dental age estimation comparisons. Dental age was estimated using the definitions of developmental stage by Moorrees, Fanning, and Hunt (1963) and the QMUL London Dental Atlas (AlQahtani et al., 2010). The skeletal age of the individuals was estimated using a combination of epiphyseal fusion data (Scheuer & Black, 2000), long bone diaphyseal lengths (Buikstra & Ubelaker, 1994; Gindhart, 1973; Maresh, 1955), and cortical bone thickness indices of the femora (Mays, 1999). The latter was carried out at the University of Bradford by taking antero‐posterior radiographs of each bone, measuring total bone width (*T*) and medullary width (*M*) and calculating the cortical index (100 × [*T* − *M*]/*T*). This index is a measure of the appositional growth of the bone and reflects the deposition of new bone (Mays, 1999).

## 3 RESULTS

### 3.1 Isotope data

The isotopic data from this study are shown in Table 2. The in utero dentine collagen sample values ranged for δ^15^N from 12.2‰ to 15.6‰, and for δ^13^C the range was from −19.8‰ to −18.4‰. All but one of the δ^15^N in utero values was higher than the maximum value from the adult bone collagen which was 12.5‰, and 12 of the 18‰ δ^13^C values were higher than the maximum adult bone collagen value of −19.4‰ (Haydock et al., 2013).

**Table 2:** Isotope data and collagen quality indicators for dentine sections from teeth from Raunds‐Furnell

Skeletal number	δ15N‰	δ13C ‰	Amt%N	Amt%C	*C*:*N*
R5093‐1	10.9	−20.1	15.4	42.5	3.2
R5093‐2	10.1	−20.0	14.7	40.6	3.2
R5093‐3	10.1	−19.7	15.0	41.0	3.2
R5093‐4	10.1	−19.6	14.8	40.3	3.2
R5093‐5	10.0	−19.5	14.6	39.8	3.2
R5093‐6	9.9	−19.3	15.1	41.4	3.2
R5093‐7	9.9	−19.4	15.6	42.7	3.2
R5093‐8	10.0	−19.5	15.2	41.8	3.2
R5093‐9	10.1	−19.6	15.1	41.6	3.2
R5093‐10	10.1	−19.5	14.9	40.8	3.2
R5093‐11	10.0	−19.5	15.0	40.7	3.2
R5093‐12	10.2	−19.5	15.4	42.4	3.2
R5093‐13	10.5	−19.4	15.0	41.3	3.2
R5093‐14	11.0	−19.4	15.0	41.3	3.2
R5093‐15	11.2	−19.6	15.5	42.9	3.2
R5093‐16	11.2	−19.8	15.1	41.5	3.2
R5154‐1	14.6	−18.9	15.4	42.0	3.2
R5154‐2	13.7	−19.0	14.9	40.7	3.2
R5154‐3	12.9	−19.1	15.7	42.8	3.2
R5154‐4	11.6	−19.6	15.3	41.8	3.2
R5154‐5	11.2	−19.8	15.5	42.3	3.2
R5154‐6	11.1	−19.7	15.6	42.4	3.2
R5154‐7	11.1	−19.6	15.1	41.2	3.2
R5154‐8	11.2	−19.6	15.2	41.4	3.2
R5154‐9	11.4	−19.6	15.7	43.1	3.2
R5154‐10	11.8	−19.4	15.4	42.2	3.2
R5154‐11	11.3	−19.5	15.3	41.9	3.2
R5154‐12	10.6	−19.7	14.9	40.7	3.2
R5154‐13	10.3	−19.8	15.3	41.6	3.2
R5154‐14	10.4	−19.7	15.0	40.5	3.2
R5154‐15	10.3	−19.7	15.5	42.4	3.2
R5154‐16	10.6	−19.6	15.3	42.0	3.2
R5135‐1	12.9	−19.6	38.0	13.8	3.2
R5135‐2	14.1	−19.1	41.1	15.0	3.2
R5135‐3	14.4	−18.9	41.6	15.3	3.2
R5135‐4	13.5	−19.0	41.3	15.2	3.2
R5135‐5	12.6	−19.3	41.7	15.2	3.2
R5135‐6	12.2	−19.3	41.2	14.9	3.2
R5135‐7	12.2	−19.3	41.5	15.1	3.2
R5135‐8	11.9	−19.4	41.4	15.1	3.2
R5135‐9	11.5	−19.7	41.6	15.0	3.2
R5135‐10	11.5	−19.7	41.5	14.7	3.3
R5135‐11	11.5	−19.6	41.2	14.7	3.3
R5135‐12	11.5	−19.6	41.1	14.5	3.3
R5212‐1	12.2	−19.4	41.2	15.1	3.2
R5212‐2	12.9	−19.0	42.2	15.5	3.2
R5212‐3	13.6	−19.1	42.0	15.5	3.2
R5212‐4	13.7	−19.1	41.6	15.3	3.2
R5212‐5	13.8	−19.3	42.2	15.5	3.2
R5212‐6	14.0	−19.4	42.1	15.4	3.2
R5212‐7	13.5	−19.4	42.0	15.2	3.2
R5212‐8	12.9	−19.5	41.9	15.0	3.3
R5212‐9	12.4	−19.5	42.0	15.3	3.2
R5212‐10	11.6	−19.6	42.2	15.3	3.2
R5212‐11	10.9	−19.8	42.0	15.1	3.2
R5212‐12	10.8	−19.8	42.1	15.2	3.2
R5212‐13	11.4	−20.1	42.3	15.2	3.2
R5264‐1	13.4	−18.5	41.7	15.2	3.2
R5264‐2	13.5	−18.4	42.0	15.3	3.2
R5264‐3	14.2	−18.5	42.0	15.2	3.2
R5264‐4	14.9	−18.8	41.6	14.8	3.3
R5264‐5	15.0	−19.0	42.1	15.0	3.3
R5264‐6	15.1	−19.0	42.0	14.9	3.3
R5264‐7	14.9	−19.1	42.1	15.1	3.3
R5264‐8	14.7	−19.3	42.0	14.9	3.3
R5264‐9	14.6	−19.3	41.8	14.8	3.3
R5264‐10	14.4	−19.6	41.8	15.0	3.2
R5345‐1	15.2	−19.8	41.4	14.9	3.2
R5345‐2	15.5	−19.5	41.6	15.0	3.2
R5345‐3	15.6	−19.5	41.3	14.9	3.2
R5345‐4	15.1	−19.7	41.5	15.0	3.2
R5345‐5	14.0	−19.8	41.1	14.7	3.3
R5345‐6	12.6	−19.8	41.3	14.8	3.2
R5345‐7	12.2	−19.9	41.4	14.9	3.2
R5345‐8	11.3	−19.9	41.9	15.3	3.2
R5345‐9	10.8	−19.9	41.4	14.9	3.2
R5345‐10	10.9	−19.8	42.1	15.3	3.2
	13.3				
R5125‐1	15.0	−18.9	22.7	8.3	3.2
R5125‐2	15.8	−18.7	41.5	15.4	3.2
R5125‐3	16.4	−18.6	41.8	15.4	3.2
R5125‐4	16.4	−18.6	40.0	14.7	3.2
R5125‐5	15.8	−18.6	41.3	15.4	3.1
R5125‐6	15.2	−18.7	41.4	15.3	3.2
R5125‐7	14.8	−18.8	41.6	15.4	3.2
R5125‐8	14.5	−18.9	41.5	15.3	3.2
R5125‐9	14.3	−19.2	42.0	15.6	3.1
R5125‐10	13.2	−19.3	41.4	15.3	3.2
R5125‐11	12.7	−19.4	41.8	15.2	3.2
R5125‐12	12.7	−19.7	41.6	14.9	3.3
R5170‐1	14.1	−18.8	42.0	15.5	3.2
R5170‐2	14.5	−18.6	41.3	15.1	3.2
R5170‐3	15.1	−18.4	40.3	15.0	3.1
R5170‐4	15.5	−18.4	41.4	15.3	3.2
R5170‐5	15.6	−18.7	41.4	15.2	3.2
R5170‐6	15.6	−18.8	41.4	15.2	3.2
R5170‐7	14.9	−19.1	41.3	15.1	3.2
R5170‐8	14.3	−19.2	40.7	14.9	3.2
R5170‐9	14.1	−19.2	41.2	15.2	3.2
R5170‐10	13.9	−19.3	40.5	14.8	3.2
R5170‐11	13.7	−19.4	40.9	14.9	3.2
R5170‐12	13.3	−19.4	40.7	14.5	3.3
R5273‐1	13.2	−18.8	42.5	15.6	3.2
R5273‐2	13.9	−18.6	41.2	15.2	3.2
R5273‐3	14.0	−18.6	41.2	15.2	3.2
R5273‐4	14.4	−18.7	41.4	15.3	3.2
R5273‐5	14.8	−19.0	41.7	15.4	3.2
R5273‐6	14.4	−19.4	41.1	15.1	3.2
R5273‐7	14.1	−19.5	41.5	15.2	3.2
R5273‐8	14.2	−19.5	41.5	15.3	3.2
R5273‐9	13.2	−19.9	41.2	15.0	3.2
R5273‐10	12.2	−20.1	41.3	14.8	3.3
R5273‐11	11.9	−20.3	41.7	14.9	3.3
R5292‐1	13.8	−19.8	40.8	14.7	3.2
R5292‐2	14.2	−19.3	41.2	15.2	3.2
R5292‐3	14.8	−19.3	41.2	15.2	3.2
R5292‐4	15.2	−19.4	41.0	15.1	3.2
R5292‐5	15.4	−19.5	40.9	15.0	3.2
R5292‐6	15.5	−19.7	40.9	15.1	3.2
R5292‐7	15.3	−19.7	41.2	15.2	3.2
R5292‐8	15.4	−19.8	41.2	15.2	3.2
R5292‐9	15.1	−19.9	41.1	15.2	3.2
R5292‐10	14.6	−20.0	40.9	15.0	3.2
R5292‐11	14.0	−20.1	40.8	15.1	3.2
R5292‐12	13.2	−19.9	41.2	15.2	3.2
R5187‐1	13.6	−19.0	14.4	40.3	3.3
R5187‐2	12.4	−19.0	14.8	40.2	3.2
R5187‐3	11.7	−19.3	14.8	40.3	3.2
R5187‐4	12.0	−19.4	14.8	40.1	3.2
R5187‐5	11.4	−19.4	15.1	40.8	3.2
R5187‐6	10.6	−19.5	15.5	41.0	3.1
R5187‐7	9.9	−19.5	15.4	41.4	3.1
R5187‐8	9.4	−19.5	15.2	40.6	3.1
R5187‐9	9.8	−19.5	15.1	40.4	3.1
R5187‐10	9.7	−19.5	15.2	40.8	3.1
R5187‐11	9.3	−19.5	14.8	39.9	3.2
R5187‐12	9.2	−19.5	14.9	40.2	3.2
R5187‐13	9.0	−19.6	14.9	40.5	3.2
R5187‐14	9.0	−19.7	15.1	40.6	3.1
R5187‐15	9.1	−19.8	15.0	40.4	3.1
R5187‐16	9.4	−19.8	15.1	40.8	3.2
R5187‐17	9.7	−19.9	14.9	40.0	3.1
R5187‐18	12.3	−19.4	14.8	40.4	3.2
R5187‐19	9.6	−19.9	15.0	40.2	3.1
R5187‐20	9.7	−19.5	14.8	40.1	3.2
R5239‐1	13.9	−19.4	15.0	40.4	3.2
R5239‐2	12.5	−19.4	15.1	40.2	3.1
R5239‐3	11.8	−19.4	15.3	40.9	3.1
R5239‐4	11.3	−19.5	15.2	40.7	3.1
R5239‐5	11.3	−19.4	15.2	40.9	3.1
R5239‐6	11.8	−19.5	15.3	40.8	3.1
R5239‐7	12.0	−19.6	15.3	40.9	3.1
R5239‐8	12.2	−19.8	15.3	41.0	3.1
R5239‐9	12.2	−19.7	15.2	41.0	3.1
R5239‐10	11.9	−19.5	15.2	41.1	3.1
R5239‐11	11.6	−19.4	15.1	40.8	3.2
R5239‐12	11.5	−19.4	15.0	40.8	3.2
R5239‐13	11.3	−19.4	15.2	40.9	3.1
R5239‐14	11.3	−19.4	15.2	41.4	3.2
R5239‐15	11.6	−19.5	15.3	41.3	3.2
R5239‐16	11.5	−19.4	15.0	40.7	3.2
R5239‐17	11.7	−19.5	15.2	41.2	3.2
R5239‐18	9.6	−19.9	15.1	40.9	3.2
	11.7				
R5021‐1	13.8	−19.5	14.7	41.2	3.3
R5021‐2	12.9	−19.9	14.9	41.0	3.2
R5021‐3	12.6	−20.2	14.9	41.0	3.2
R5021‐4	12.5	−20.4	15.1	41.2	3.2
R5021‐5	11.9	−20.0	15.0	40.8	3.2
R5021‐6	11.5	−19.7	15.0	40.9	3.2
R5021‐7	10.7	−19.6	15.2	41.1	3.2
R5021‐8	10.6	−19.7	15.0	40.8	3.2
R5021‐9	10.6	−19.9	14.9	40.6	3.2
R5021‐10	10.5	−20.0	15.0	41.0	3.2
R5021‐11	10.5	−20.1	15.0	40.8	3.2
R5021‐12	10.5	−20.1	14.9	41.0	3.2
R5021‐13	10.0	−20.1	14.7	40.7	3.2
R5021‐14	10.0	−20.1	14.8	40.7	3.2
R5021‐15	10.3	−20.1	14.6	40.6	3.2
R5021‐16	10.4	−19.9	14.5	40.7	3.3
R5338‐1	13.9	−18.6	14.9	40.9	3.2
R5338‐2	13.1	−18.5	15.2	41.5	3.2
R5338‐3	13.6	−18.6	15.0	40.7	3.2
R5338‐4	12.3	−18.7	15.0	40.5	3.2
R5338‐5	10.7	−18.9	15.2	41.1	3.2
R5338‐6	8.5	−19.6	15.0	40.9	3.2
R5338‐7	7.6	−19.7	15.0	40.9	3.2
R5338‐8	7.4	−19.6	15.0	41.0	3.2
R5338‐9	7.9	−19.6	15.1	40.9	3.2
R5338‐10	8.4	−19.8	14.8	40.6	3.2
R5338‐11	9.1	−20.1	14.7	40.3	3.2
R5338‐12	9.5	−19.7	14.7	40.5	3.2
R5338‐13	9.3	−19.6	14.7	40.7	3.2
R5338‐14	8.5	−19.8	14.7	40.8	3.2
R5338‐15	8.6	−19.5	14.5	40.4	3.3
R5338‐16	9.2	−19.6	14.3	39.5	3.2
R5012‐1	13.8	−18.4	41.6	15.3	3.2
R5012‐2	14.5	−18.4	40.9	14.9	3.2
R5012‐3	14.9	−18.5	41.7	15.2	3.2
R5012‐4	15.2	−18.7	41.9	15.3	3.2
R5012‐5	15.3	−18.8	41.6	15.2	3.2
R5012‐6	15.0	−19.1	41.2	15.1	3.2
R5012‐7	14.8	−19.3	44.1	15.9	3.2
R5012‐8	14.2	−19.4	43.4	15.9	3.2
R5012‐9	14.0	−19.5	45.9	16.9	3.2
R5012‐10	13.5	−19.7	41.9	15.4	3.2
R5012‐11	13.5	−19.9	42.0	15.4	3.2
R5012‐12	13.3	−20.0	43.1	15.9	3.2
R5012‐13	13.0	−20.1	42.3	15.5	3.2
R5251‐1	12.6	−19.7	41.2	14.8	3.3
R5251‐2	12.5	−19.7	43.2	15.6	3.2
R5251‐3	12.6	−19.4	41.4	14.8	3.3
R5251‐4	12.8	−19.3	42.1	15.1	3.2
R5140‐1	15.4	−19.0	40.7	14.8	3.2
R5140‐2	15.6	−18.8	43.0	15.8	3.2
R5140‐3	15.9	−18.7	40.9	15.1	3.2
R5140‐4	16.1	−18.7	40.7	15.0	3.2
R5140‐5	16.2	−18.7	40.0	14.7	3.2
R5140‐6	16.6	−18.8	41.1	15.1	3.2
R5140‐7	17.1	−19.2	42.7	15.5	3.2
R5354‐1	15.6	−19.2	41.1	14.9	3.2
R5354‐2	16.6	−18.7	40.8	15.1	3.2
R5354‐3	17.6	−18.5	40.9	15.1	3.2
R5354‐4	17.9	−18.8	41.2	15.2	3.2
R5354‐5	17.5	−19.0	41.0	15.1	3.2
R5354‐6	17.5	−19.0	41.2	15.2	3.2
R5354‐7	16.4	−19.0	41.5	15.2	3.2
R5354‐8	14.4	−19.5	41.0	14.6	3.3
R5354‐9	12.8	−19.9	41.2	14.6	3.3
R5354‐10	11.9	−20.2	40.5	14.4	3.3
R5102‐1	14.4	−18.9	35.5	12.8	3.2
R5102‐2	15.2	−18.6	41.2	15.2	3.2
R5102‐3	15.9	−18.6	41.1	15.2	3.2
R5102‐4	16.3	−18.7	41.8	15.5	3.1
R5102‐5	16.5	−18.7	41.6	15.1	3.2
R5102‐6	16.4	−18.8	41.7	15.1	3.2
R5102‐7	16.3	−18.9	41.6	15.1	3.2
R5102‐8	16.0	−19.2	41.9	15.0	3.2
R5102‐9	15.4	−19.4	41.3	14.8	3.3
R5102‐10	14.6	−19.5	41.2	14.7	3.3
R5102‐11	14.1	−19.5	41.1	14.7	3.3
R5102‐12	12.8	−19.5	41.2	14.8	3.3
R5082‐1	13.5	−19.1	30.1	10.8	3.2
R5082‐2	14.0	−18.9	40.8	15.1	3.2
R5082‐3	14.6	−18.8	40.5	14.8	3.2
R5082‐4	15.5	−18.9	39.7	14.3	3.2
R5082‐5	16.5	−18.8	39.8	14.5	3.2
R5082‐6	17.4	−18.9	40.7	15.1	3.2
R5005‐1	14.5	−19.1	43.9	15.9	3.2
R5005‐2	14.5	−19.2	41.7	15.3	3.2
R5005‐3	15.1	−19.2	41.8	15.3	3.2
R5005‐4	15.1	−19.4	41.9	15.2	3.2
R5005‐5	14.6	−19.8	41.9	15.1	3.2
R5005‐6	14.0	−20.0	41.9	14.8	3.3
R5005‐7	13.1	−20.2	42.1	14.9	3.3
R5005‐8	13.0	−20.3	41.6	14.6	3.3
R5005‐9	12.4	−20.3	42.1	14.7	3.3
R5005‐10	12.1	−20.5	41.9	14.8	3.3
R5005‐11	11.8	−20.2	41.0	14.6	3.3
R5005‐12	11.4	−20.2	41.0	14.6	3.3
R5023‐1	13.2	−19.4	66.7	23.9	3.3
R5023‐2	14.7	−19.1	41.7	15.2	3.2
R5023‐3	15.1	−19.0	42.0	15.6	3.1
R5023‐4	15.4	−18.9	42.4	15.7	3.1
R5023‐5	15.4	−19.0	42.0	15.6	3.1
R5023‐6	15.4	−19.0	41.9	15.4	3.2
R5023‐7	14.8	−19.1	42.2	15.5	3.2
R5023‐8	14.0	−19.4	41.9	15.0	3.3
R5023‐9	13.2	−19.8	41.9	14.9	3.3
R5023‐10	12.5	−19.9	41.8	14.8	3.3
R5023‐11	11.9	−19.8	39.8	14.4	3.2
R5023‐12	11.5	−19.8	41.3	14.5	3.3
R5070‐1	13.5	−18.8	40.6	14.8	3.2
R5070‐2	12.7	−19.3	41.0	14.7	3.3
R5070‐3	13.2	−19.1	40.7	14.5	3.3
R5070‐4	13.7	−18.9	41.4	14.7	3.3
R5070‐5	14.0	−18.9	39.7	14.0	3.3
R5070‐6	14.3	−19.0	41.0	14.5	3.3
R5070‐7	14.4	−19.1	39.7	13.8	3.3
R5070‐8	14.5	−19.0	38.2	13.3	3.4
R5070‐9	14.1	−19.1	37.8	13.0	3.4
R5070‐10	13.9	−19.3	35.3	12.0	3.4
R5070‐11	13.6	−19.5	31.2	10.6	3.4
R5070‐12	13.6	−19.6	32.4	10.8	3.5
R5070‐13	13.4	−19.5	34.9	11.9	3.4
R5109‐1	15.5	−18.7	48.9	17.7	3.2
R5109‐2	14.0	−18.7	41.8	15.3	3.2
R5109‐3	14.8	−18.5	41.8	15.3	3.2
R5109‐4	15.3	−18.4	42.1	15.5	3.2
R5109‐5	15.6	−18.5	42.1	15.4	3.2
R5109‐6	15.8	−18.4	42.1	15.4	3.2
R5109‐7	16.0	−18.1	41.8	15.4	3.2
R5109‐8	15.9	−18.2	42.1	15.6	3.2
R5109‐9	16.2	−18.1	42.1	15.4	3.2
R5109‐10	15.9	−18.0	42.1	15.6	3.2
R5109‐11	15.5	−18.0	42.1	15.5	3.2
R5109‐12	15.2	−18.1	42.0	15.5	3.2
R5109‐13	14.9	−18.3	41.9	15.5	3.2
R5109‐14	14.5	−18.5	42.6	15.6	3.2
R5109‐15	13.6	−18.6	42.3	15.6	3.2

The highest peak dentine δ^15^N value from the profiles was R5354, 17.9‰, and the lowest was R5093, 11.2‰. The highest peak dentine δ^13^C was R5109, −18.0‰, and the lowest was R5021, −19.5‰.

Across all the juvenile dentine profiles the maximum variation in δ^13^C values across the life course was 1.5‰ for R5338. The maximum variation in δ^15^N across the life course for deciduous teeth was 6.0‰ (R5354), but for the M1 from R5338 was 6.5‰.

### 3.2 Skeletal and dental ages

All of the juveniles re‐assessed (*n* = 15) had a dental age between birth and 9 years, and a skeletal age between birth and 7 years. A total of 79% of the individuals showed a higher dental age than skeletal age, 13% had a higher skeletal age than dental age and the remaining 8% had matching skeletal and dental ages. The difference between the two age estimations increased with age suggesting a cumulative effect with continued survival (this trend was also seen when Raunds was compared to other archaeological populations in the study by Lewis (2002)). The largest difference was found in R5338 with a dental age of 9 years and a skeletal age of only 6 years.

## 4 DISCUSSION

### 4.1 Isotopic data from comparative sites

**Figure 2:**
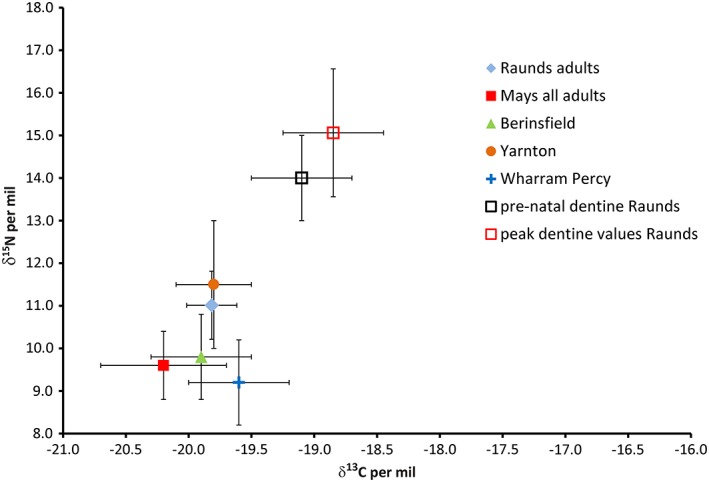
Biplot showing the carbon (δ^13^C) and nitrogen (δ^15^N) isotope ratios for mean bulk bone collagen for 5 Anglo‐Saxon British sites compared with prenatal dentine collagen and peak dentine collagen mean δ^13^C and δ^15^N. Sites shown are Raunds (*n* = 20; Haydock et al. 2013), early Anglo Saxons (*n* = 76; Mays and Beavan 2012, Wharram Percy (*n* = 29; Richards et al. 2002), Berinsfield adults (*n* = 65; Privat et al., 2002), Yarnton (*n* = 9; Lightfoot et al., 2009) [Color figure can be viewed at http://wileyonlinelibrary.com]

Figure 2 shows the mean values for adult bone collagen δ^13^C and δ^15^N for 4 comparative Anglo‐Saxon skeletal samples, alongside bone collagen data from adults from Raunds Furnells (Haydock et al., 2013) and the means for both pre‐natal and peak dentine measurements of deciduous dentine from this study. Sites shown are Raunds (*n* = 20; Haydock et al., 2013), Early Anglo‐ Saxons (*n* = 76; Mays & Beavan, 2012), Wharram Percy (*n* = 29; Richards, Mays, & Fuller, 2002), Berinsfield adults (*n* = 65; Privat, O'Connell, & Richards, 2002), Yarnton (*n* = 9; Lightfoot et al., 2009). The isotopic data from Raunds Furnells suggests that the adult population were not consuming a different diet from contemporary populations which could explain the peri‐natal dentine values.

### 4.2 Peri‐natal and peak dentine collagen data

It can be seen that the dentine collagen δ^13^C and δ^15^N are already higher than the Raunds adult mean by 0.7‰ and 3‰ at birth, which is equivalent to the differences interpreted as a trophic level shift and a breastfeeding signal by most studies which measure bulk bone (e.g., Fuller et al., 2006; Fuller, Richards, & Mays, 2003; Haydock et al., 2013; Jay, 2005; Figure 2). As this represents the δ^13^C and δ^15^N in utero, it also reflects the maternal values during the third trimester of pregnancy. This could imply that women during pregnancy were consuming foods which differ from the usual inland Anglo‐Saxon diet (for example marine foods). Isotopic evidence from sequentially‐forming tissues such as hair and fingernail show that healthy modern women during pregnancy generally experience a slight reduction in δ^15^N (e.g., Fuller et al., 2006; D'Ortenzio, Brickley, Schwarcz, & Prowse, 2015) due to the anabolic state induced by the pregnancy. The women of Raunds Furnells could also have been experiencing physiological and/or nutritional stress resulting in a catabolic state, as seen in the case of severe morning sickness in a modern pregnancy by Fuller, Fuller, Sage, Harris, and O'Connell (2005), and thus the high δ^15^N values found in the in utero dentine, (and see discussion in Reitsema (2013) although this does not explain the raised δ^13^C). However, maternal physiology changes during pregnancy as a result of fat storage and transfer to the fetus (and see above) which may explain the perinatal δ^13^C. If these maternal changes in δ^13^C and δ^15^N are short‐term (during pregnancy and breastfeeding) they will not be visible in the bone collagen at a population level because of the effect of averaging and bone turnover.

The peak dentine δ^13^C and δ^15^N (which in the bone collagen weaning model by Millard (2000) would represent the age at which exclusive breastfeeding ceased and weaning began) is 4.1‰ higher for δ^15^N and 1‰ higher for δ^13^C than the Raunds adult mean. This difference is considerably more than the 2–3‰ increase in bone collagen δ^15^N during exclusive breastfeeding reported in the review by Tsutaya and Yoneda (2015), which suggests that this is not a simple “trophic level” effect. The offset between the paired juvenile bone collagen data (Haydock et al., 2013) and the highest dentine data ranges from 0.3 to 4.5‰, with the dentine values for each individual higher than the bone.

When comparing peak δ^15^N of dentine and bulk juvenile bone collagen, it appears the collagen is recording different values in the two tissues of the same individual. To explore this offset, the dentine profile data are discussed further in the next section.

### 4.3 Dentine profiles

The dentine profiles were very variable. For four of the five adults there was an identifiable drop in δ^15^N with a co‐varying drop in δ^13^C from the earliest age measured, which could be interpreted as a weaning curve. R5093 was the exception, with a short 0.8 per mil drop in δ^15^N between the first and second sections (at about 18 months of age), but with the δ^13^C values rising from the earliest section until the age of 4 years. In all the adult profiles, there was overlap between the juvenile (dentine) δ^15^N values and the adult bone collagen values, suggesting some continuity between the diet and physiology throughout life (see Supporting Information Figures).

Most of the juvenile δ^15^N profiles also showed a pattern consistent with a weaning curve, with the δ^15^N rising from the in utero section, reaching a peak and then dropping back, generally to a lower value than at birth. In some cases, the δ^13^C values fall much earlier than the δ^15^N. Two (R5070, R5109) have a drop in the δ^15^N from the in utero value to the first post‐natal value, and then follow a weaning curve pattern. R5140, R5082, R5251 (a neonate) and R5264 all have very flat or diverging δ^13^C and δ^15^N profiles.

**Figure 3:**
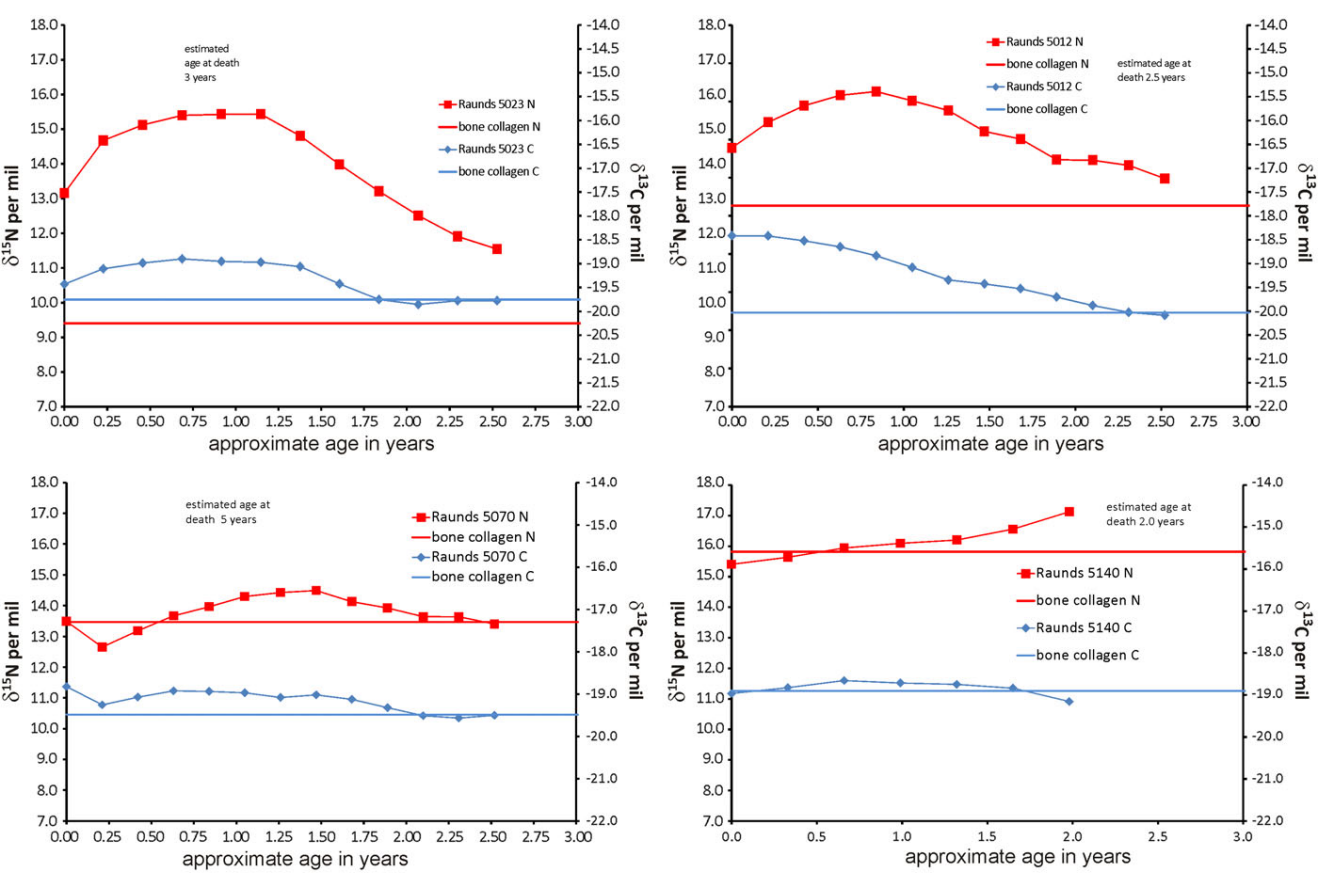
Incremental dentine carbon (δ^13^C) and nitrogen (δ^15^N) isotope ratio profiles by estimated age for deciduous teeth from Raunds, R5023, R5012, R5070, and R5140 as exemplars of main patterns seen in deciduous dentine profiles at this site [Color figure can be viewed at http://wileyonlinelibrary.com]

Four main patterns have been identified in these data (Figure 3 shows exemplars), for which interpretations based on dietary and physiological status can be provided as follows:

### 4.4 Pattern 1: Weaning curve (R5023)

In these individuals, the δ^13^C and δ^15^N values in the dentine profiles co‐vary and match the weaning curve model proposed by previous papers based on the assessment of bone collagen (e.g., Jay, 2005; Millard, 2000). This is interpreted as a dietary signal consistent with the expected pattern of breastfeeding, complementary feeding and weaning, with no evidence for overlying physiological effects on δ^15^N. (this pattern also seen in R5135, R5125, R5354).

### 4.5 Pattern 2: Weaning curve with overlying physiological effects (R5012)

The dentine collagen profiles show a smooth weaning curve in the δ^13^C values, but the δ^15^N remains high for an extended period. This suggests that there is a secondary factor which is affecting the δ^15^N values. This factor is not likely to be dietary, as any rise in trophic level arising from dietary protein input would also result in a comparable elevation of δ^13^C (also seen in R5005, R5102, R5170, R5212, R5273, R5292, R5345). There could be an unknown factor affecting the δ^13^C but a viable explanation for the rise in δ^15^N without δ^13^C is the effects of physiological stress.

### 4.6 Pattern 3: Sharp drop in δ^13^C and δ^15^N values followed by pattern 1 or 2 (R5070)

These infants (R5070 and R5109) experience δ^15^N and δ^13^C values prior to birth that are elevated in comparison to their post‐natal values. The former, which reflect in utero experience, could arise from maternal physiological stress impacting on the developing fetus. The initially elevated levels would then fall after birth as the infants’ dietary input becomes the main source of δ^15^N and δ^13^C.

### 4.7 Pattern 4: Flat or rising δ^13^C and δ^15^N profiles (R5140)

In these individuals (R5140, R5082, R5251, R5264), the flat δ^13^C and δ^15^N profiles suggest that they had little or no breastmilk. R5264 (see Supporting Information figures) has opposing covariance of the δ^13^C and δ^15^N values, resembling the starvation patterns seen in the incremental dentine of children from the Great Irish Famine (Beaumont & Montgomery, 2016) and Sumburgh cist (Montgomery et al., 2013).

In the case of R5251, aged as a fetus/neonate, the sections of dentine lining the enamel‐dentine junction were all co‐forming in utero with no overlap/averaging and because they did not survive birth, no dietary signal.

### 4.8 Comparison of bone collagen and dentine collagen δ^13^C and δ^15^N

One of the aims of this study was to explore the relationship between bone and dentine collagen values from single individuals. As bone is constantly turning over during life, there will be a time‐averaging of isotope values over the remodeling period. This averaging also occurs in infant bone collagen where turnover is rapid, albeit to a lesser extent than in adult bone where turnover is slower. In comparison, the averaging of isotope values in the dentine sections is much less, and the first 0.5 mm sample should reflect the in utero period.

**Figure 4:**
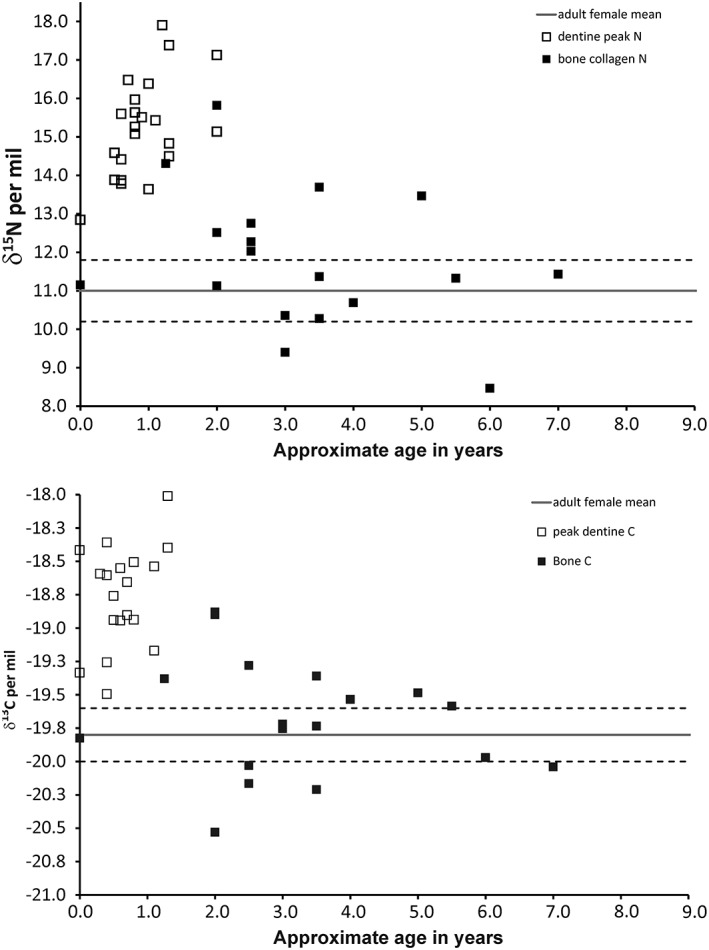
Carbon (δ^13^C) and nitrogen (δ^15^N) isotope ratio of bone collagen and peak dentine collagen from Raunds juveniles plotted against mean age in years. Solid lines are the mean, and dashed lines 1 standard deviation of bone collagen δ^13^C and δ^15^N values of Raunds adult females of child‐bearing age (bone collagen data from Haydock et al. 2013)

Figure 4 shows both the bone collagen values from the study by Haydock et al. (2013), and the peak dentine values from this study, using the same individuals. If the model by Millard (2000) is applied, then the bone collagen δ^15^N values suggest that exclusive breastfeeding in the population ceases at about 2 years. The peak dentine δ^15^N values (which should therefore represent the commencement of weaning in each individual using the same model) suggest that these individuals are in fact reaching that point between 6 and 18 months of age, with two commencing weaning at the age of 2 years. The peak δ^13^C values in the dentine (Figure 4) suggest an even earlier introduction of cereal‐based weaning foods with all individuals peaking before 18 months of age. The δ^13^C should be a more robust representation of the diet as the trophic level shift would be much smaller, and values are potentially less affected by nutritional stress.

These data call into question the validity of certain interpretations of the bone collagen data. If the dentine collagen data reflects diet alone, then they must be more accurate measures of the actual commencement of weaning in an individual. The two collagen datasets do not match, and there is a much higher difference between most of the dentine collagen δ^13^C and δ^15^N values and the maternal mean than in the bone collagen data.

**Figure 5:**
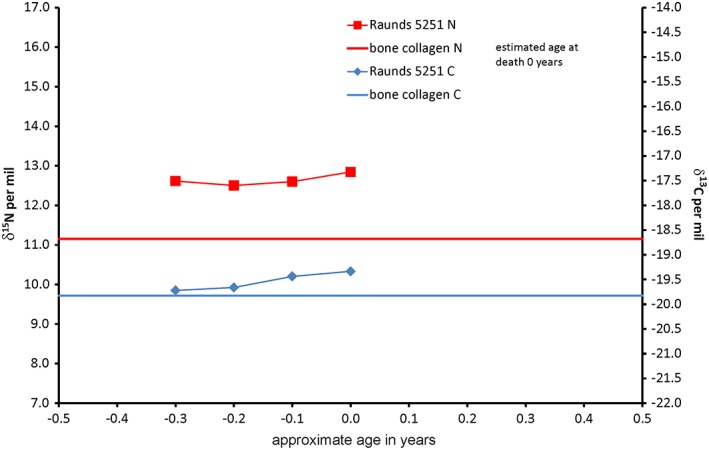
Incremental dentine carbon (δ^13^C) and nitrogen (δ^15^N) isotope ratio profile for neonate Raunds5251 [Color figure can be viewed at http://wileyonlinelibrary.com]

**Figure 6:**
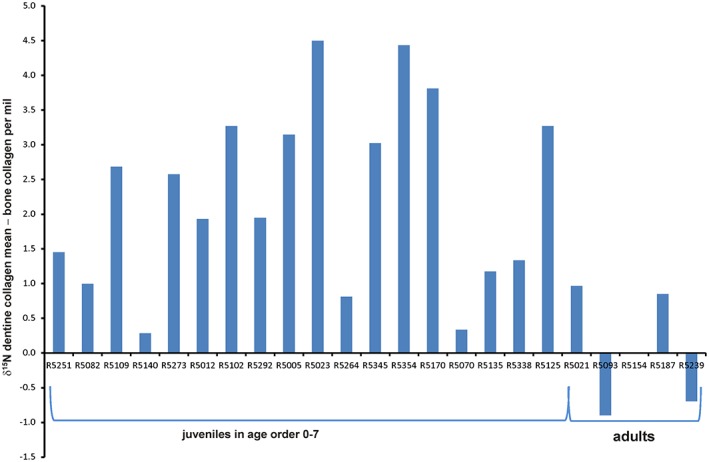
Plot showing mean dentine collagen nitrogen (δ^15^N) isotope ratios – bulk bone collagen nitrogen (δ^15^N) isotope ratios per mil (‰) for Raunds juveniles and adults in age order [Color figure can be viewed at http://wileyonlinelibrary.com]

As can be seen from the dentine profiles, in some cases the bone collagen δ^15^N values do not overlap with the dentine collagen profile. This is especially marked with R5251, the neonate: the bone and dentine must have been co‐forming in utero and yet the bone collagen δ^15^N is over 1‰ lower (Figure 5). Figure 6 shows the difference between the bone collagen and the mean dentine δ^15^N for all individuals in this study. As the bone represents an average value for δ^13^C and δ^15^N over the same period as tooth formation in the younger juveniles it could be assumed that the values for the two tissues should be the same. However, the youngest eight individuals have mean dentine δ^15^N values which are higher than their bone by 0.3 to 3.3‰.

Although there are very few comparisons of deciduous incremental dentine and bone collagen, this offset has been recorded in other datasets. Eight out of nine deciduous teeth analyzed from 19th century Lukin Street, London (Beaumont, 2013) have no overlap and an offset with dentine collagen δ^15^N higher than the bulk bone collagen. The datasets from late Medieval Fishergate House, York, (thought to be a low status population) show similar patterns to the Raunds Furnells data (Burt, 2013, 2015). Of the juveniles sampled at Fishergate House, 23 were under 3.5 years of age and had measurements for late‐forming dentine which should be co‐forming with their rib collagen. A comparison of the measurements shows that 6 of the 23 had δ^15^N rib collagen values which do not overlap with any of their dentine values and are between 2.2 and 0.2‰ lower than the latest‐forming dentine. This offset was also evident in the data from the recent publication by King et al. (2018) where five of the eight matched dentine/bulk bone pairs have δ^15^N values that do not overlap: four have higher dentine collagen δ^15^N and one lower. This appears to contradict the conclusions of King et al. (2018) that bulk bone collagen can be used to reconstruct breastfeeding and weaning behavior.

One potential explanation is that there is an offset between collagen in dentine and bone or that the bone is not recording the highest δ^15^N values. There is no evidence to suggest that there is any difference in the proteins in type 1 collagen between dentine and bone and so these values must be reflecting the δ^13^C and δ^15^N in new tissue laid down by dentinoblasts or osteoblasts although the routing of the amino acids to create the new collagen protein may be different. An alternative explanation is that there is a threshold of stress above which osteoblasts do not produce any new bone collagen, while dentinoblasts continue to produce dentine which records different values for δ^13^C and δ^15^N. This would fit well with the concept that bone and thus skeletal growth is salutatory and the gaps between episodes of growth are initiated by nutritional, physiological and emotional stress followed by periods of catch‐up growth once the stress is reduced, thus recording δ^13^C and δ^15^N at these lower levels only (Lampl, 2012). Neonate R5251 demonstrates this offset which must be caused by high levels of maternal and/or fetal stress in utero. The relationship between high δ^15^N and stunting is explored below.

Where weaning studies have been produced from bone collagen isotope data alone, conclusions are most often supported by the δ^15^N data, while the δ^13^C values are either unreported or not discussed in as much detail. The dentine collagen profiles presented in this study suggest that, while the magnitude of changes in values are small, δ^13^C appears to reflect a breastfeeding and weaning profile in more cases. The δ^13^C values are also less affected by any physiological changes than δ^15^N and thus a more robust measure of the expected changes seen during breastfeeding and weaning (although δ^13^C may fall if body fat is being recycled during periods of starvation; Beaumont & Montgomery, 2016). A further tentative explanation is that maternal physiology in the later stages of breastfeeding includes the mobilization of fat stores and a fall in the δ^13^C of breastmilk and thus the infant tissues as seen in the example by Herrscher et al. (2017).

In all 18 juveniles, the dentine profiles have reached their peak δ^13^C values by the age of 1.3 years (±3 months) and, where individuals survive past the age of 3, match their bulk bone collagen values by the age of 2.5–3 years. This results in agreement with the interpretations based on bone collagen for the same population in Haydock et al. (2013): an exclusive breastfeeding period of about 1 year and cessation of breastfeeding at about 2.5–3 years. However, nearly half of the peak dentine collagen δ^15^N (8/18) values are later than 1 year of age, and the peaks for two individuals are at the age of 2 years. Interpreting these high values using the bone collagen model, the δ^15^N data would imply that exclusive breastfeeding had continued until the age of 2, which would have been both unlikely and unhealthy. However, we could hypothesize that the δ^15^N values are also reflecting increased stress at this period of life, so recording the diet *plus* recycled nitrogen. Thus, while we appear to have identified a period of breastfeeding and weaning in this population, we know that 4/18 apparently did not receive breastmilk, and 8/18 have isotopic evidence for physiological stress after the proposed introduction of weaning foods. This means that 2/3 of the juveniles analyzed do not appear to have had a weaning curve that matches the model (Jay, 2005; Millard, 2000) reinforcing the view that this is not a satisfactory way of estimating this aspect of cultural behavior.

### 4.9 The relationship between stunting and δ^13^C and δ^15^N

**Figure 7:**
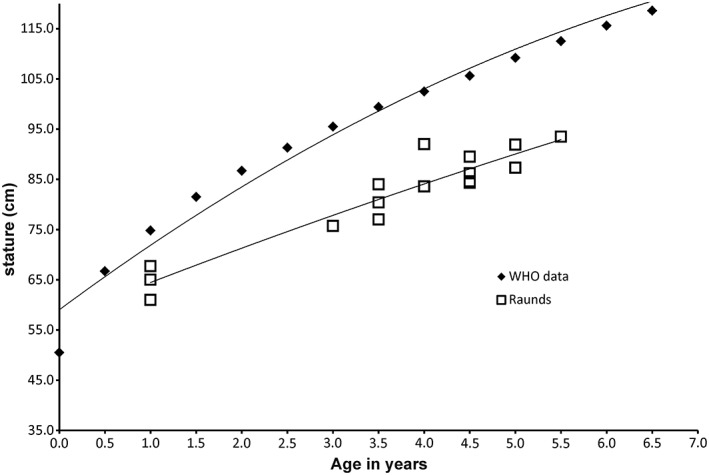
Plot showing polynomial trends for growth for World Health Organization stature tables (Cole et al., 1998) and estimated stature of juveniles from Raunds (*n* = 15)

**Figure 8:**
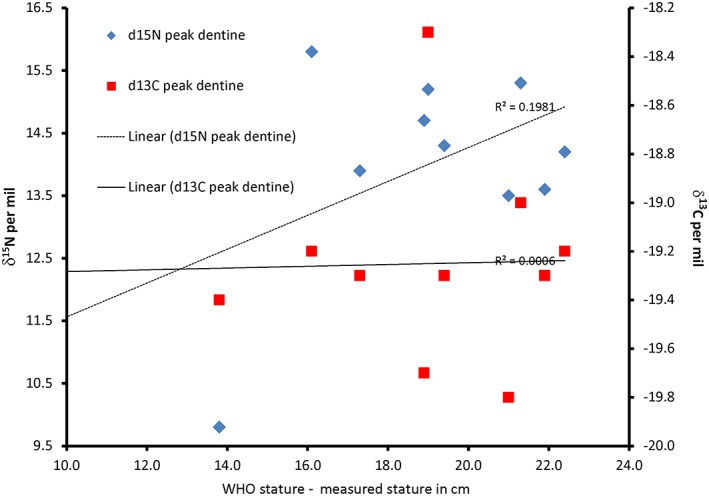
Plot showing the relationship between peak dentine carbon (δ^13^C) and nitrogen (δ^15^N) isotope ratios and stunting of individuals from Raunds as measured by the difference between World Health Organization stature tables for modern English children (Cole et al., 1998) and stature estimated from skeletal remains [Color figure can be viewed at http://wileyonlinelibrary.com]

For the purposes of this study, we estimated skeletal and dental ages (see results and Table 3) and also used long bone measurements to estimate stature of nine of the juveniles. Figure 7 compares the achieved stature for the re‐assessed Raunds juveniles, and demonstrates the cumulative effect of stunting, that is the Raunds juveniles diverge more from the WHO growth stature standards as age increases. Figure 8 shows the difference between current WHO data (Cole, Freeman, & Preece, 1998) and the measured stature at Raunds Furnells plotted against the peak values recorded in the dentine for both the δ^13^C and δ^15^N. As stated above, the difference between dental and skeletal age rose with overall age and this cumulative stunting was also evident in the stature differences. There is a positive correlation between the level of stunting and δ^15^N (*R* ^2^ = 0.1981) which is not evident in the δ^13^C (*R* ^2^ = 0.0006).

**Table 3:** Estimated skeletal age, dental age, and stature for juveniles from Raunds–Furnell

Skeletal number	Skeletal age (in years)	Dental age (in years)	Estimated stature (in cm)	WHO standard ‐measured stature (in cm)
R5005	3.5	4.5	84.6	21.0
R5012	2.0	3.5	77.0	22.4
R5023	3.0	5.0	91.9	17.3
R5102	2.0	4.5	84.3	21.3
R5109	2.5	3.5	80.4	19.0
R5140	0.5	1.0	65.0	9.8
R5233	4.0	5.5	93.5	19.0
R5251	2.0	1.0	67.7	7.1
R5271	3.5	4.5	86.2	19.4
R5273	2.0	5.0	87.3	21.9
R5292	2.5	4.0	83.6	18.9
R5302	1.0	3.5	84.0	15.4
R5310	1.5	3.0	75.7	19.8
R5329	3.0	4.0	92.0	10.5
R5338	0.5	1.0	61.0	13.8
R5354	3.0	4.5	89.5	16.1

It must be borne in mind that the peak values for δ^13^C and δ^15^N occurred during the formation of the teeth, before the age of 3 years in all nine cases, yet the level of stunting recorded related to the age at death which for many of the individuals was several years later. This suggests that stress during the first years of life is a predictor of stunting that continues later into childhood. This helps to corroborate the earlier finding that the dentine collagen values appear to record higher short‐term δ^15^N than is visible in the bone collagen δ^15^N, and supports the hypothesis that bone is not laid down during periods of high stress which results in a long‐lasting effect on the stature of the individual.

## 5 CONCLUSIONS

This study is the first to compare isotopic data from bone and dentine collagen in the same individuals to investigate the relationship between diet, physiology, and stunting in the early years of life. The improved temporal resolution achieved using incremental dentine, already seen in previous studies, has now been reinforced by the possibility that bone is not forming during extreme stress and thus is not a reliable source of dietary OR physiological information in a stressed juvenile. However, it may be useful to consider the δ^13^C values for estimation of breastfeeding and weaning patterns as these appear more robust especially as most weaning foods appear to be cereal‐based, low trophic‐level proteins (although the recent paper by Herrscher et al. (2017) hints at a possible physiological explanation here too). This confirms earlier work (e.g., Beaumont et al., 2015) that suggested that bulk bone collagen is not the right tissue to utilize when investigating the breastfeeding and weaning period because of the influence of physiology, particularly on the δ^15^N values. These data also reinforce the need to consider the “osteological paradox” (DeWitte & Stojanowski, 2015; Wood et al., 1992) before interpreting the isotope ratios from juvenile tissues.

The relationship between maternal and infant in utero δ^13^C and δ^15^N values requires further investigation to establish whether the differences between these are related to a special pregnancy diet, stress of mother or fetus, or another factor of which we are not aware.

Finally, there is a need to investigate the potential for δ^15^N as a tool to measure the effect of stress in early life on growth and stunting during childhood and adolescence, and to develop methods which can collect data from bone with increased temporal resolution matching that of the dentine collagen.

## Supporting information

Figure S1. Incremental dentine carbon (δ^13^C) and nitrogen (δ^15^N) isotope ratio profiles by estimated age for deciduous teeth from Raunds matching profile type 1

Figure S2. (a,b) Incremental dentine carbon (δ^13^C) and nitrogen (δ^15^N) isotope ratio profiles by estimated age for deciduous teeth from Raunds matching profile type 2

Figure S3. Incremental dentine carbon (δ^13^C) and nitrogen (δ^15^N) isotope ratio profiles by estimated age for deciduous tooth from Raunds matching profile type 3

Figure S4. Incremental dentine carbon (δ^13^C) and nitrogen (δ^15^N) isotope ratio profiles by estimated age for deciduous teeth from Raunds matching profile type 2

Figure S5 (a,b) Incremental dentine carbon (δ^13^C) and nitrogen (δ^15^N) isotope ratio profiles by estimated age for permanent teeth from Raunds demonstrating variable profiles including flat (R5093), co‐varying (R5154) opposing co‐variance (R5021 and R5235) and wide range of variable values (R5187 and R5338).

## References

[ajpa23682-bib-0001] AlQahtani, S. J. , Hector, M. P. , & Liversidge, H. M. (2010). Brief communication: The London Atlas of Human Tooth development and eruption. American Journal of Physical Anthropology, 142, 481–490. 20310064 10.1002/ajpa.21258

[ajpa23682-bib-0002] AlQahtani, S. J. , Hector, M. P. , & Liversidge, H. M. (2014). Accuracy of dental age estimation charts: Schour and Massler, Ubelaker and the London atlas. American Journal of Physical Anthropology, 154(1), 70–78. 10.1002/ajpa.22473 24470177

[ajpa23682-bib-0003] Armit, I. , Shapland, F. , Montgomery, J. , & Beaumont, J. (2015). Difference in death? A lost Neolithic inhumation cemetery with Britain's earliest case of rickets, at Balevullin, Western Scotland. Proceedings of the Prehistoric Society., 81, 199–214.

[ajpa23682-bib-0004] Beaumont, J. (2013). *An isotopic and historical study of diet and migration during the Great Irish potato famine 1845‐1852* (PhD PhD), University of Bradford, Bradford.

[ajpa23682-bib-0005] Beaumont, J. , Gledhill, A. , Lee‐Thorp, J. , & Montgomery, J. (2013). Childhood diet: A closer examination of the evidence from dental tissues using stable isotope analysis of incremental human dentine. Archaeometry, 55(2), 277–295. 10.1111/j.1475-4754.2012.00682.x

[ajpa23682-bib-0006] Beaumont, J. , Gledhill, A. , & Montgomery, J. (2014). Isotope analysis of incremental human dentine: Towards higher temporal resolution. Bulletin of the International Association for Palaeodontology, 8(2), 212–223.

[ajpa23682-bib-0007] Beaumont, J. , & Montgomery, J. (2016). The great Irish famine: Identifying starvation in the tissues of victims using stable isotope analysis of bone and incremental dentine collagen. PLoS One, 11(8), e0160065 10.1371/journal.pone.0160065 27508412 PMC4980051

[ajpa23682-bib-0008] Beaumont, J. , Montgomery, J. , Buckberry, J. , & Jay, M. (2015). Infant mortality and isotopic complexity: New approaches to stress, maternal health, and weaning. American Journal of Physical Anthropology, 157(3), 441–457. 10.1002/ajpa.22736 25773670

[ajpa23682-bib-0009] Boddington, A. (1996). Raunds Furnells. The Anglo‐Saxon church and churchyard. London, UK: English Heritage.

[ajpa23682-bib-0010] Bose, A. (2018). Let us talk about stunting. Journal of Tropical Pediatrics, 104, 174–175. 10.1093/tropej/fmx104 29315413

[ajpa23682-bib-0011] Brown, T. A. , Nelson, D. E. , Vogel, J. S. , & Southon, J. R. (1988). Improved collagen extraction by modified Longin method. Radiocarbon, 30, 171–177.

[ajpa23682-bib-0012] Buikstra, J. E. , & Ubelaker, D. H. (1994). Standards for data collection from human remains (Vol. 44). Fayetteville, AR: Arkansas Archaeological Survey.

[ajpa23682-bib-0013] Burt, N. M. (2013). Stable isotope ratio analysis of breastfeeding and weaning practices of children from medieval Fishergate house York, UK. American Journal of Physical Anthropology, 152(3), 407–416. 10.1002/ajpa.22370 24105083

[ajpa23682-bib-0014] Burt, N. M. (2015). Individual dietary patterns during childhood: An archaeological application of a stable isotope microsampling method for tooth dentin. Journal of Archaeological Science, 53, 277–290. 10.1016/j.jas.2014.10.019

[ajpa23682-bib-0015] Butte, N. F. , Hopkinson, J. M. , Wong, W. W. , Smith, E. O. B. , & Ellis, K. J. (2000). Body composition during the first 2 years of life: An updated reference. Pediatric Research, 47, 578–585. 10813580 10.1203/00006450-200005000-00004

[ajpa23682-bib-0072] Cameron, N. (2012). The Curve of Human Growth In Cameron N. & Bogin B. (Eds.) Human Growth and Development (2nd ed.) London: Academic Press.

[ajpa23682-bib-0016] Cherel, Y. , Hobson, K. A. , Bailleul, F. , & Groscolas, R. (2005). Nutrition, physiology, and stable isotopes: New information from fasting and molting penguins. Ecology, 86(11), 2881–2888.

[ajpa23682-bib-0017] Cole, T. J. , Freeman, J. V. , & Preece, M. A. (1998). British 1990 growth reference centiles for weight, height, body mass index and head circumference fitted by maximum penalized likelihood. Statistical Medicine, 17(4), 407–429. 9496720

[ajpa23682-bib-0018] Craig, E. (2005). *An osteological and palaeopathological assessment of stress indicators and social status at Raunds Furnells, Northamptonshire*. (unpublished MSc dissertation), University of Bradford, Bradford.

[ajpa23682-bib-0019] Craig, E. F. , & Buckberry, J. L. (2010). Investigating social status using evidence of biological status: A case study from Raunds Furnells In Buckberry J. L. & Cherryson A. K. (Eds.), Burial in later Anglo‐Saxon England, c.650–1,100 AD (pp. 128–142). Oxford, UK: Oxbow.

[ajpa23682-bib-0020] de Luca, A. , Boisseau, N. , Tea, I. , Louvet, I. , Robins, R. J. , Forhan, A. , … Hankard, R. (2012). δ15N and δ13C in hair from newborn infants and their mothers: A cohort study. Pediatric Research, 71, 598–604. 22398698 10.1038/pr.2012.3

[ajpa23682-bib-0021] DeNiro, M. (1987). Stable isotopy and archaeology. American Scientist, 75, 182–191.

[ajpa23682-bib-0074] D'Ortenzio, L. , Brickley, M. , Schwarcz, H. P. & Prowse, T. (2005). You are not what you eat during physiological stress: isotopic evaluation of human hair. American Journal of Physical Anthropology, 157, 374–388. 10.1002/ajpa.22722 25711625

[ajpa23682-bib-0022] DeWitte, S. , & Stojanowski, C. (2015). The osteological paradox 20 years later: Past perspectives, Future Directions. Journal of Archaeological Research, 23, 1–54.

[ajpa23682-bib-0023] Elamin, F. , & Liversidge, H. M. (2013). Malnutrition has no effect on the timing of human tooth formation. PLoS One, 8(8), e72274. 24023614 10.1371/journal.pone.0072274 PMC3758289

[ajpa23682-bib-0024] Floud, R. , Wachter, K. , & Gregory, A. (1990). Height, health and history. Cambridge, UK: Cambridge University Press.

[ajpa23682-bib-0025] Fogel, M. , Tuross, N. , & Owsley, D. (1989). Nitrogen isotope tracers of human lactation in modern and archaeological populations. Carnegie Institite of Washington Yearbook, 88, 111–117.

[ajpa23682-bib-0075] Fuller, B. T. , Fuller, J. L. , Sage, N. E. , Harris, D. A. , O'Connell, T. C. & Hedges, R. E. M. (2005). Nitrogen balance and δ15N: why you're not what you eat during nutritional stress. Rapid Communications in Mass Spectrometry, 19(18), 2497–2506. 16106342 10.1002/rcm.2090

[ajpa23682-bib-0026] Fuller, B. T. , Fuller, J. L. , Harris, D. A. , & Hedges, R. E. M. (2006). Detection of breastfeeding and weaning in modern human infants with carbon and nitrogen stable isotope ratios. American Journal of Physical Anthropology, 129(2), 279–293. 10.1002/ajpa.20249 16261548

[ajpa23682-bib-0027] Fuller, B. T. , Richards, M. , & Mays, S. A. (2003). Stable carbon and nitrogen isotope variations in tooth dentine serial sections from Wharram Percy. Journal of Archaeological Science, 30, 1673–1684.

[ajpa23682-bib-0028] Gindhart, P. S. (1973). Growth standards for the tibia and radius in children ages one month through eighteen years. American Journal of Physical Anthropology, 39(1), 41–48. 4351576 10.1002/ajpa.1330390107

[ajpa23682-bib-0029] Hadley, D. M. , & Buckberry, J. L. (2005). Caring for the dead in late Anglo‐Saxon England In Tinti F. (Ed.), Pastoral care in late Anglo‐Saxon England (pp. 121–147). Woodbridge, ON: Boydell.

[ajpa23682-bib-0030] Haydock, H. , Clarke, L. , Craig‐Atkins, E. , Howcroft, R. , & Buckberry, J. (2013). Weaning at Anglo‐Saxon Raunds: Implications for changing breastfeeding practice in Britain over two Millenia. American Journal of Physical Anthropology, 151, 604–612. 23868173 10.1002/ajpa.22316

[ajpa23682-bib-0031] Henderson, R. C. , Lee‐Thorp, J. , & Loe, L. (2014). Early life histories of the London poor using δ13C and δ15N stable isotope incremental dentine sampling. American Journal of Physical Anthropology, 154(4), 585–593. 10.1002/ajpa.22554 24898314

[ajpa23682-bib-0032] Herrscher, E. , Goude, G. , & Metz, L. (2017). Longitudinal study of stable isotope compositions of maternal milk and implications for the Palaeo‐diet of infants. BMSAP Bulletins et mémoires de la Société d'anthropologie de Paris., 29, 131–139. 10.1007/s13219-017-0190-4

[ajpa23682-bib-0033] Hoppa, R. D. (1992). Evaluating human skeletal growth: An Anglo‐Saxon example. International Journal of Osteoarchaeology, 2(4), 275–288. 10.1002/oa.1390020403

[ajpa23682-bib-0034] Howcroft, R. (2008). *The interaction between diet, disease and stable isotopes: a study of bone and teeth from the Anglo‐Saxon cemetery at Raunds Furnells*. (Unpublished MSc dissertation), University of Bradford, Bradford, UK.

[ajpa23682-bib-0035] Howcroft, R. , Eriksson, G. , & Lidén, K. (2012). Conformity in diversity? Isotopic investigations of infant feeding practices in two iron age populations from southern Öland, Sweden. American Journal of Physical Anthropology, 149(2), 217–230. 22826010 10.1002/ajpa.22113

[ajpa23682-bib-0036] Ives, R. (2015). Insights into health, life and death in Victorian London's east end. London Archaeologist, 14, 150–154.

[ajpa23682-bib-0037] Jantz, R. L. , & Owsley, D. W. (1984). Long bone growth among Arikara skeletalmpopulations. American Journal of Physical Anthropology, 63, 13–20. 6703031 10.1002/ajpa.1330630103

[ajpa23682-bib-0038] Jay, M. (2005). *Stable isotope evidence for British Iron Age diet: Inter‐ and intra‐site variation in carbon and nitrogen from bone collagen at Wetwang in East Yorkshire and sites in East Lothian, Hampshire and Cornwall*. (PhD thesis). University of Bradford, Bradford.

[ajpa23682-bib-0039] Jay, M. , Fuller, B. T. , Richards, M. P. , Knüsel, C. J. , & King, S. S. (2008). Iron age breastfeeding practices in Britain: Isotopic evidence from Wetwang slack, East Yorkshire. American Journal of Physical Anthropology, 136, 327–337. 18324632 10.1002/ajpa.20815

[ajpa23682-bib-0040] Katzenberg, M. A. , & Lovell, N. C. (1999). Stable isotope variation in pathological bone. International Journal of Osteoarchaeology, 9, 316–324.

[ajpa23682-bib-0041] King, C. L. , Millard, A. R. , Gröcke, D. R. , Standen, V. G. , Arriaza, B. T. , & Halcrow, S. E. (2018). A comparison of using bulk and incremental isotopic analyses to establish weaning practices in the past. STAR: Science & Technology of Archaeological Research, 3, 126–134. 10.1080/20548923.2018.1443548

[ajpa23682-bib-0042] Lampl, M. (2012). Saltation and stasis In Cameron N. & Bogin B. (Eds.), Human growth and development (2nd ed.). London, UK: Academic Press.

[ajpa23682-bib-0043] Larsen, C. S. (2015). Bioarchaeology, interpreting behavior from the human skeleton in (2nd ed.). Cambridge: Cambridge University Press.

[ajpa23682-bib-0044] Lehn, C. , Rossmann, A. , & Graw, M. (2015). Provenancing of unidentified corpses by stable isotope techniques‐ presentation of case studies. Science & Justice, 55(1), 72–88. 10.1016/j.scijus.2014.10.006 25577010

[ajpa23682-bib-0045] Lejarraga, H. (2012). Growth in infancy and childhood: A pediatric approach In Cameron N. & Bogin B. (Eds.), Human growth and development (2nd ed.). London, UK: Academic Press.

[ajpa23682-bib-0046] Lewis, M. (2002). Impact of industrialization: Comparative study of child health in four sites from medieval and postmedieval England (A.D. 850–1859). American Journal of Physical Anthropology, 119, 211–223. 12365033 10.1002/ajpa.10126

[ajpa23682-bib-0047] Lewis, M. E. (2007). The bioarchaeology of children: Perspectives from biological and forensic anthropology. Cambridge, UK: Cambridge University Press.

[ajpa23682-bib-0048] Lightfoot, E. , O'Connell, T. C. , Stevens, R. E. , Hamilton, J. , Hey, G. , & Hedges, R. (2009). An investigation into diet at the site of Yarnton, Oxfordshire, using stable carbon and nitrogen isotopes. Oxford Journal of Archaeology, 28, 301–322.

[ajpa23682-bib-0049] Maresh, M. (1955). Liner growth of the long‐bones of the extremities from infancy through adolescence. American Journal of Diseases of Children, 89(6), 725–742. 14375378

[ajpa23682-bib-0073] Mays, S. , Brickley, M. , Ives, R. (2008). Growth in an English population from the Industrial Revolution. American Journal of Physical Anthropology, 136(1), 85–92. 18186509 10.1002/ajpa.20780

[ajpa23682-bib-0050] Mays, S. (1999). Linear and appositional long bone growth in earlier human populations: A case study from mediaeval England In Hoppa R. D. & Fitzgerald C. M. (Eds.), Human growth in the past: Studies from bones and teeth (pp. 290–312). Cambridge, UK: Cambridge University Press.

[ajpa23682-bib-0051] Mays, S. (2016). Estimation of stature in archaeological human skeletal remains from Britain. American Journal of Physical Anthropology, 161(4), 646–655. 10.1002/ajpa.23068 27535104

[ajpa23682-bib-0052] Mays, S. , & Beavan, N. (2012). An investigation of diet in early Anglo‐Saxon England using carbon and nitrogen stable isotope analysis of human bone collagen. Journal of Archaeological Science, 39(4), 867–874.

[ajpa23682-bib-0053] Millard, A. R. (2000). A model for the effect of weaning on nitrogen isotope ratios in humans In Goodfriend G. A., Collins M. J., Fogel M., Macko S. A., & Wehmiller J. F. (Eds.), Perspectives in amino acid and protein geochemistry. Oxford, UK: Oxford University Press.

[ajpa23682-bib-0054] Montgomery, J. , Beaumont, J. , Jay, M. , Keefe, K. , Gledhill, A. R. , Cook, G. T. , … Melton, N. D. (2013). Strategic and sporadic marine consumption at the onset of the Neolithic: Increasing temporal resolution in the isotope evidence. Antiquity, 87(338), 1060–1072.

[ajpa23682-bib-0055] Moorrees, C. F. A. , Fanning, E. A. , & Hunt, E. E. (1963). Age variation of formation stages for ten permanent teeth. Journal of Dental Research, 42, 1490–1502. 14081973 10.1177/00220345630420062701

[ajpa23682-bib-0056] Müldner, G. , Chenery, C. , & Eckardt, H. (2011). The “headless romans”: Multi‐isotope investigations of an unusual burial ground from roman Britain. Journal of Archaeological Science, 38(2), 280–290.

[ajpa23682-bib-0057] Neuberger, F. M. , Jopp, E. , Graw, M. , Püschel, K. , & Grupe, G. (2013). Signs of malnutrition and starvation: Reconstruction of nutritional life histories by serial isotopic analyses of hair. Forensic Science International, 226(1‐3), 22–32. 10.1016/j.forsciint.2012.10.037 23374882

[ajpa23682-bib-0058] Pinhasi, R. , Teschler‐Nicola, M. , Knaus, A. , & Shaw, P. (2005). Cross‐population analysis of the growth of long bones and the os coxae of three early medieval Austrian populations. American Journal of Human Biology, 17(4), 470–488. 15981184 10.1002/ajhb.20406

[ajpa23682-bib-0059] Powell, F. (1996). The human remains In Boddington A. (Ed.), Raunds Furnells: The Anglo‐Saxon church and churchyard. London, UK: English Heritage.

[ajpa23682-bib-0060] Privat, K. , O'Connell, T. C. , & Richards, M. P. (2002). Stable isotope analysis of human and faunal remains from the Anglo‐Saxon cemetery at Berinsfield, Oxfordshire: Dietary and social implications. Journal of Archaeological Science, 29, 779–790.

[ajpa23682-bib-0061] Reitsema, L. J. (2013). Beyond diet reconstruction: Stable isotope applications to human physiology, health, and nutrition. American Journal of Human Biology, 25(4), 445–456. 10.1002/ajhb.22398 23784719

[ajpa23682-bib-0062] Ribot, I. , & Roberts, C. (1996). A study of non‐specific stress indicators and skeletal growth in two mediaeval subadult populations. Journal of Archaeological Science, 23(1), 67–79. 10.1006/jasc.1996.0006

[ajpa23682-bib-0063] Richards, M. P. , Mays, S. , & Fuller, B. T. (2002). Stable carbon and nitrogen isotope values of bone and teeth reflect weaning age at the medieval Wharram Percy site, Yorkshire, UK. American Journal of Physical Anthropology, 119(3), 205–210. 10.1002/ajpa.10124 12365032

[ajpa23682-bib-0064] Sandberg, P. A. , Sponheimer, M. , Lee‐Thorp, J. , & Van Gerven, D. (2014). Intra‐tooth stable isotope analysis of dentine: A StepToward addressing selective mortality in the reconstruction of life history in the archaeological record. American Journal of Physical Anthropology, 155, 281–293. 25156177 10.1002/ajpa.22600

[ajpa23682-bib-0065] Saunders, S. R. , & Hoppa, R. D. (1993). Growth deficit in survivors and non‐survivors: Biological mortality bias in subadult skeletal samples. American Journal of Physical Anthropology, 36(S17), 127–151. 10.1002/ajpa.1330360608

[ajpa23682-bib-0066] Scheuer, L. , & Black, S. M. (2000). Developmental juvenile osteology. San Diego, CA: Academic Press.

[ajpa23682-bib-0067] Sutphen, J. L. (1985). Growth as a measure of nutritional stress. Journal of Pediatric Gastroenterology and Nutrition, 4, 169–181. 3886867 10.1097/00005176-198504000-00004

[ajpa23682-bib-0068] Tanner, J. M. (1989). Foetus into man: Physical growth from conception to maturity. Ware, UK: Castlemead Publications.

[ajpa23682-bib-0069] Tsutaya, T. , & Yoneda, M. (2015). Reconstruction of breastfeeding and weaning practices using stable isotope and trace element analyses: A review. American Journal of Physical Anthropology, 156, 2–21. 25407359 10.1002/ajpa.22657

[ajpa23682-bib-0070] WHO . (2013). Childhood stunting: Context, causes and consequences. WHO Conceptual Framework. Geneva, Switzerland: WHO.

[ajpa23682-bib-0071] Wood, J. W. , Milner, G. R. , Harpending, H. C. , Weiss, K. M. , Cohen, M. N. , & Eisenberg, L. E. (1992). The osteological paradox: Problems of inferring prehistoric health from skeletal samples [and comments and reply]. Current Anthropology, 33(4), 343–370.

