# Supplementary figures and images for: Comparing apples and oranges: Why infant bone collagen may not reflect dietary intake in the same way as dentine collagen
Source: Am J Phys Anthropol. 2018 Sep 6;167(3):524–40. doi: 10.1002/ajpa.23682 (PMC6221104; doi:10.1002/ajpa.23682)

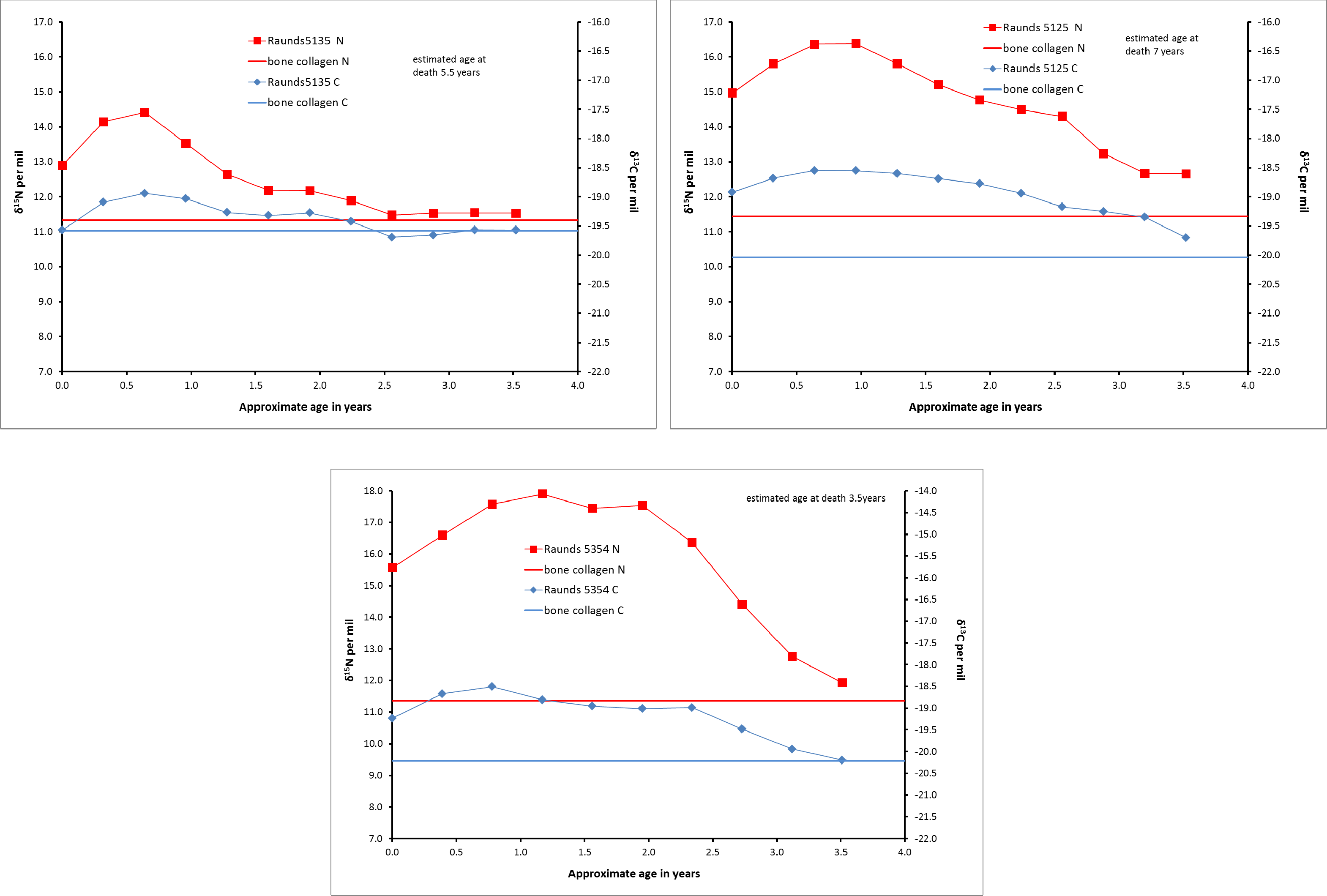

Supplement: Supplementary file 1 — Figure S1. Incremental dentine carbon (δ13C) and nitrogen (δ15N) isotope ratio profiles by estimated age for deciduous teeth from Raunds matching profile type 1 [file AJPA-167-524-s001.tif]

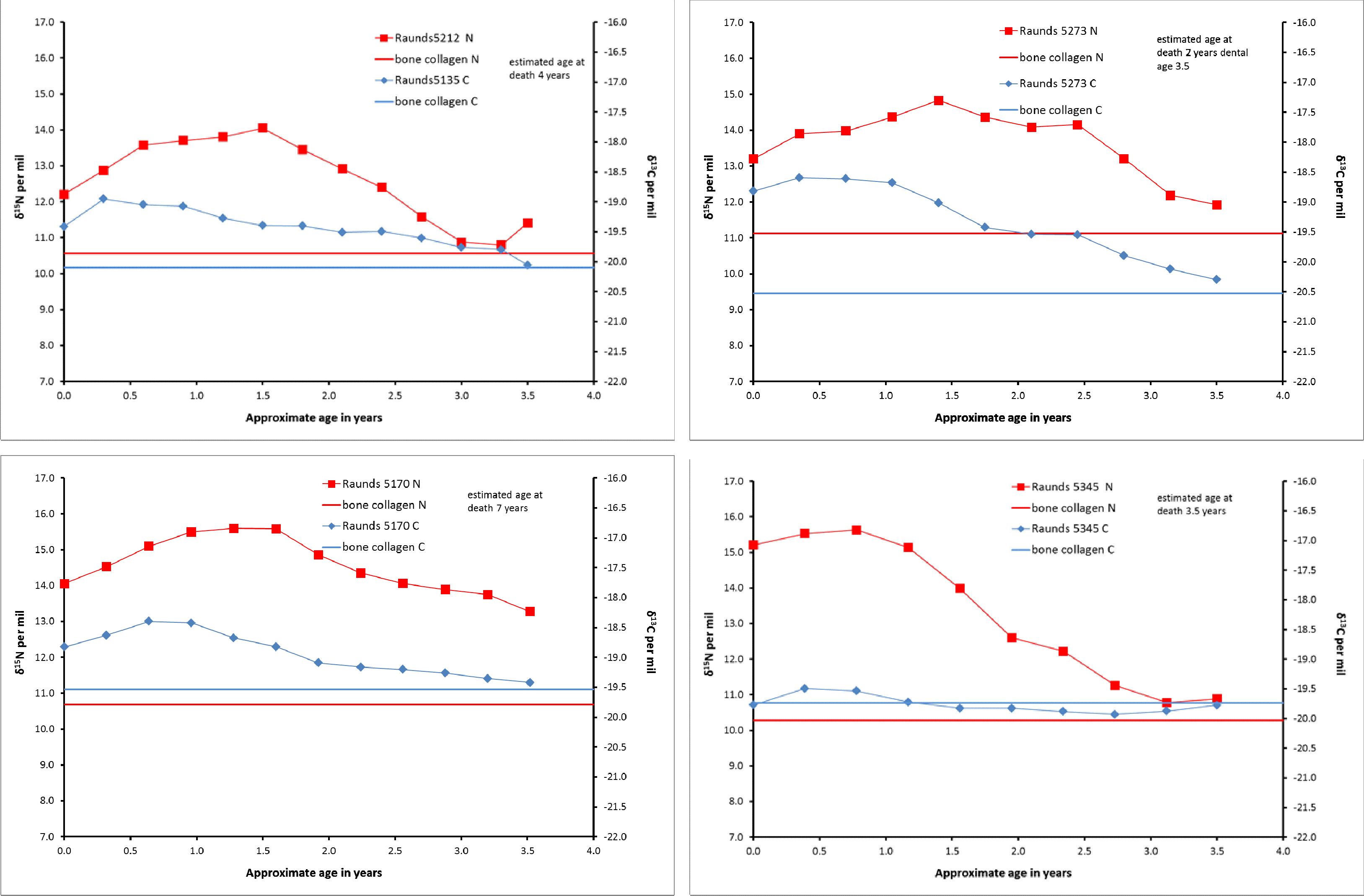

Supplement: Supplementary file 2 — Figure S2. (a,b) Incremental dentine carbon (δ13C) and nitrogen (δ15N) isotope ratio profiles by estimated age for deciduous teeth from Raunds matching profile type 2 [file AJPA-167-524-s002.zip › Figure_S2a.tif]

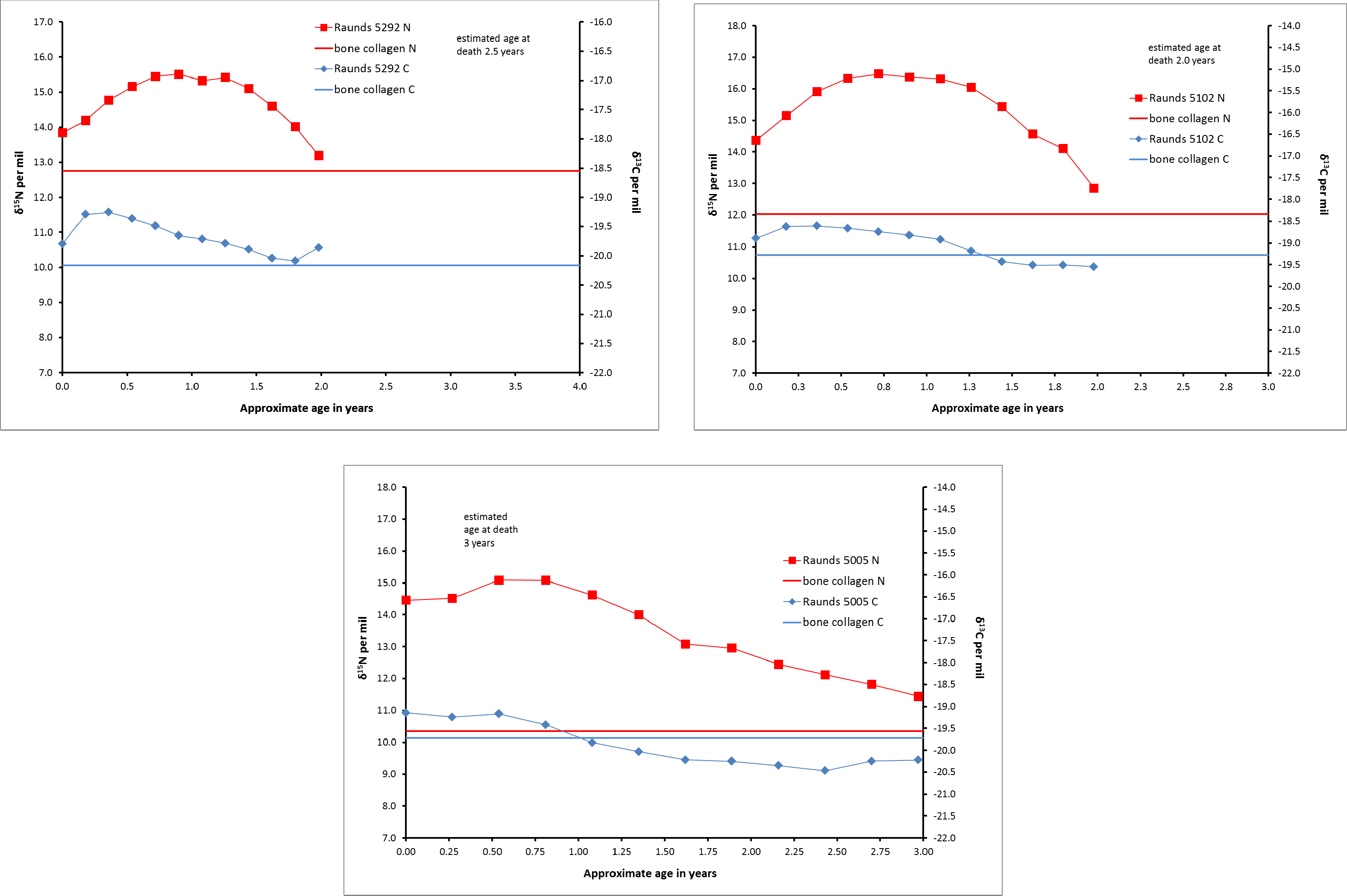

Supplement: Supplementary file 2 — Figure S2. (a,b) Incremental dentine carbon (δ13C) and nitrogen (δ15N) isotope ratio profiles by estimated age for deciduous teeth from Raunds matching profile type 2 [file AJPA-167-524-s002.zip › Figure_S2b.tif]

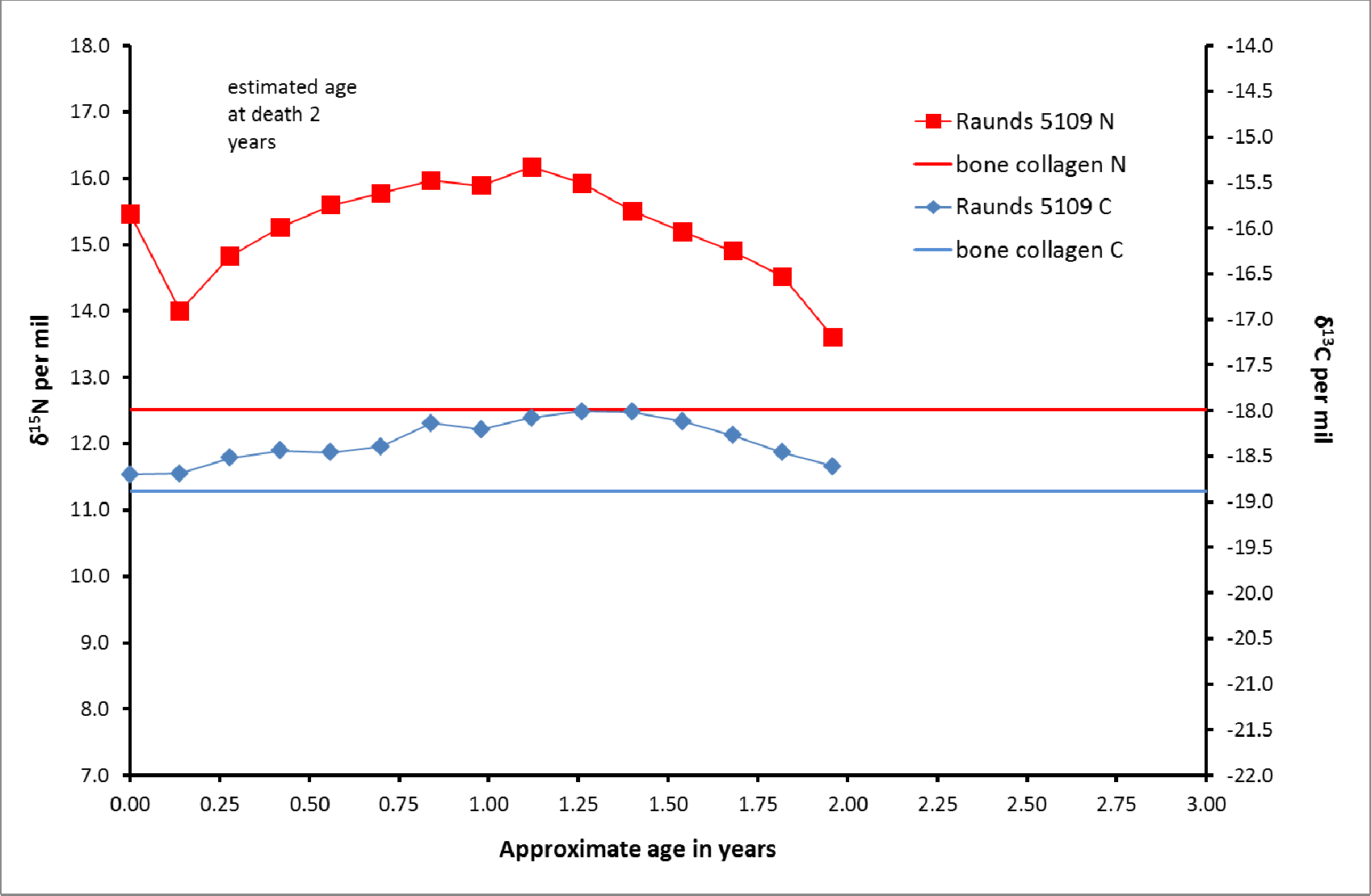

Supplement: Supplementary file 3 — Figure S3. Incremental dentine carbon (δ13C) and nitrogen (δ15N) isotope ratio profiles by estimated age for deciduous tooth from Raunds matching profile type 3 [file AJPA-167-524-s003.tif]

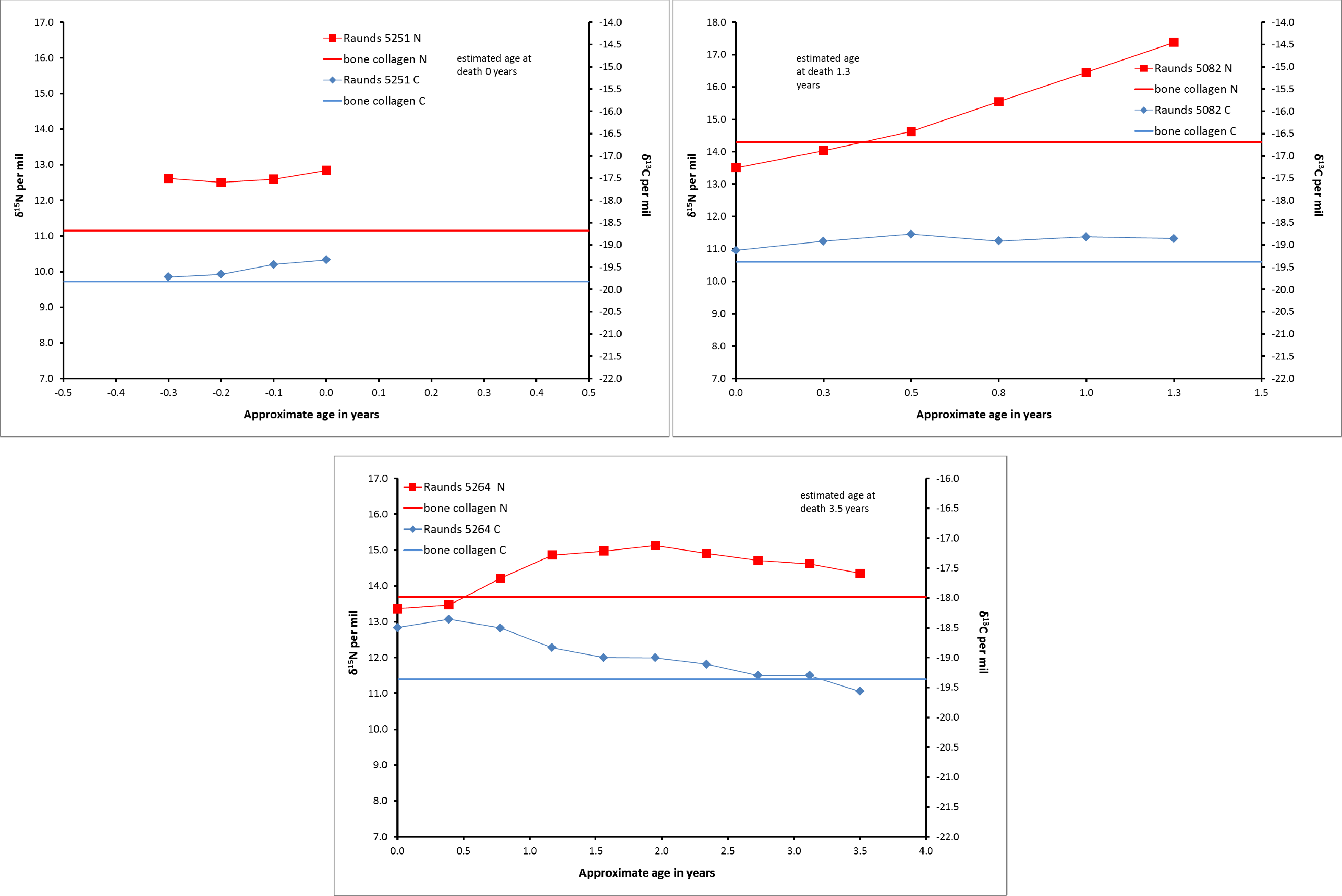

Supplement: Supplementary file 4 — Figure S4. Incremental dentine carbon (δ13C) and nitrogen (δ15N) isotope ratio profiles by estimated age for deciduous teeth from Raunds matching profile type 2 [file AJPA-167-524-s004.tif]

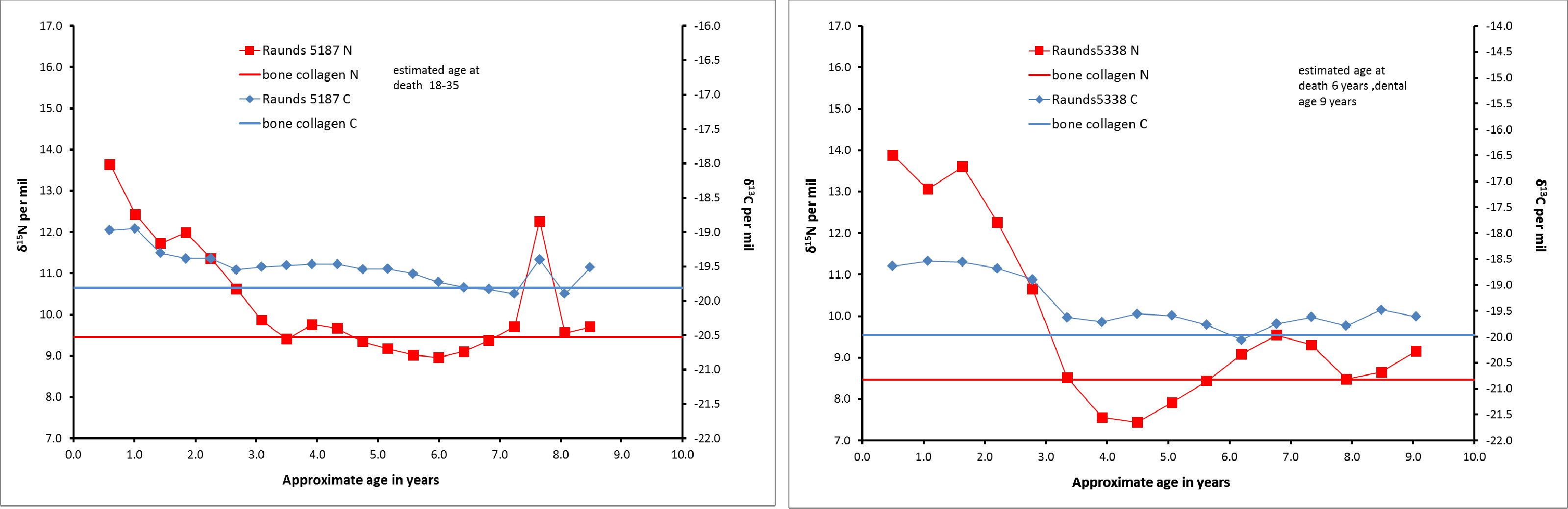

Supplement: Supplementary file 5 — Figure S5 (a,b) Incremental dentine carbon (δ13C) and nitrogen (δ15N) isotope ratio profiles by estimated age for permanent teeth from Raunds demonstrating variable profiles including flat (R5093), co‐varying (R5154) opposing co‐variance (R5021 and R5235) and wide range of variable values (R5187 and R5338). [file AJPA-167-524-s005.zip › Figure_S5b.tif]
